# Reprogramming Macrophage Polarization, Depleting ROS by Astaxanthin and Thioketal‐Containing Polymers Delivering Rapamycin for Osteoarthritis Treatment

**DOI:** 10.1002/advs.202305363

**Published:** 2023-12-14

**Authors:** Huiyun Li, Yusong Yuan, Lingpu Zhang, Chun Xu, Hailin Xu, Zhiwei Chen

**Affiliations:** ^1^ Department of Orthopedic Surgery The First Affiliated Hospital of University of South China Hengyang Hunan 421001 China; ^2^ Department of Orthopaedic Surgery China‐Japan Friendship Hospital No.2 Yinghuayuan East Street Beijing 100029 China; ^3^ Beijing National Laboratory for Molecular Science State Key Laboratory of Polymer Physics and Chemistry Institute of Chemistry Chinese Academy of Science Beijing 100190 China; ^4^ School of Dentistry The University of Queensland Brisbane 4006 Australia; ^5^ Department of Trauma and Orthopedics Peking University People's Hospital Diabetic Foot Treatment Center Peking University People's Hospital 11th XizhimenSouth Street Beijing 100044 China

**Keywords:** Astaxanthin, macrophage polarization, osteoarthritis, rapamycin, thioketal

## Abstract

Osteoarthritis (OA) is a chronic joint disease characterized by synovitis and joint cartilage destruction. The severity of OA is highly associated with the imbalance between M1 and M2 synovial macrophages. In this study, a novel strategy is designed to modulate macrophage polarization by reducing intracellular reactive oxygen species (ROS) levels and regulating mitochondrial function. A ROS‐responsive polymer is synthesized to self‐assemble with astaxanthin and autophagy activator rapamycin to form nanoparticles (NP@Poly^RHAPM^). In vitro experiments show that NP@Poly^RHAPM^ significantly reduced intracellular ROS levels. Furthermore, NP@Poly^RHAPM^ restored mitochondrial membrane potential, increased glutathione (GSH) levels, and promoted intracellular autophagy, hence successfully repolarizing M1 macrophages into the M2 phenotype. This repolarization enhanced chondrocyte proliferation and vitality while inhibiting apoptosis. In vivo experiments utilizing an anterior cruciate ligament transection (ACLT)‐induced OA mouse model revealed the anti‐inflammatory and cartilage‐protective effects of NP@Poly^RHAPM^, effectively mitigating OA progression. Consequently, the findings suggest that intra‐articular delivery of ROS‐responsive nanocarrier systems holds significant promise as a potential and effective therapeutic strategy for OA treatment.

## Graphical abstract

A novel reactive oxygen species‐responsive biodegradable nanoparticle (NP@Poly^RHAPM^) is designed, in which astaxanthin can reduce intracellular ROS levels and rapamycin‐enhanced autophagy levels, and restore mitochondrial function, hence successfully repolarizing M1 macrophages into M2 phenotypes. This repolarization enhances the proliferation and viability of chondrocytes while inhibiting apoptosis. Overall, this ROS‐responsive nanocarrier system provides a new avenue for progressive OA therapy.

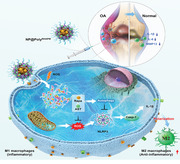

## 1 Introduction

Osteoarthritis (OA) is a chronic, low‐grade inflammatory joint disease characterized by synovitis and degeneration of joint cartilage.^[^ ^1^ ^]^ Prolonged wear and tear of the joint cartilage exacerbates synovitis, leading to joint dysfunction and a significant decline in quality of life.^[^ ^2^ ^]^ Recent studies have revealed inflammation, specifically synovitis, as a key contributing factor to the onset and progression of OA.^[^ ^3^ ^]^ Interestingly, even in the absence of cartilage damage, synovitis induced by other joint structures can ultimately lead to the development of early‐stage osteoarthritis.^[^ ^4^ ^]^ Nonsteroidal anti‐inflammatory drugs (NSAIDs) are currently widely employed for OA management, aiming to alleviate inflammation and mitigate pain. However, NSAIDs have inherent limitations such as limited bioavailability, rapid metabolism, and non‐specific targeting, which result in unfavorable side effects.^[^ ^5^ ^]^ Researchers are actively exploring novel approaches to regulate the local release of medications at the site of inflammation, prolong their residence time, and minimize drug exposure to healthy tissues. This pursuit aims to enhance the efficacy and safety of OA treatments, offering a promising avenue for future therapeutic interventions.

Numerous studies have demonstrated that the imbalance of M1/M2 type synovial macrophages plays a pivotal role in the pathogenesis and progression of OA.^[^ ^6^ ^]^ M1‐type macrophages, also known as pro‐inflammatory macrophages, exhibit a pro‐inflammatory phenotype and secrete various inflammatory substances, including interleukin‐1 β (IL‐1β), IL‐6, tumor necrosis factor‐α (TNF‐α), and nitric oxide (NO). These macrophages primarily contribute to the initiation and amplification of inflammation.^[^ ^7^ ^]^ Conversely, M2 macrophages possess an anti‐inflammatory phenotype and secrete cytokines such as IL‐10, which are crucial for vascular remodeling and tissue healing.^[^ ^8^ ^]^ In the early stages of OA inflammation, M1‐type macrophages predominate and are estimated to be 4.8 times more abundant than M2‐type macrophages.^[^ ^9^ ^]^ The excessive release of damaging cytokines and chemokines by M1 macrophages contributes to joint cartilage damage. Conversely, Dai et al. discovered that M2 macrophages immunomodulated by squid type II collagen released pro‐chondrogenic cytokines (TGF‐β1 and TGF‐β3) and prevented chondrocyte apoptosis and matrix metalloproteinase‐13 (MMP13) production, remodeling the local joint microenvironment and mediating cartilage repair in OA.^[^ ^10^ ^]^ Those data indicated that macrophages play different functions in the mediation of chondrogenesis depending on their diverse polarization phenotypes. Consequently, modulating the M2/M1 macrophage ratio represents an effective therapeutic strategy. The precise molecular mechanisms underlying the conversion of M1 macrophages to the M2 phenotype remain unknown and necessitate further investigation by researchers. Recent studies have indicated a close association between mitochondrial dysfunction and the imbalance of M1/M2 macrophages in OA.^[^ ^11^ ^]^ Notably, M1 macrophages generate excessive reactive oxygen species (ROS). Disruption of the balance between ROS production and clearance leads to elevated oxidative stress levels, resulting in mitochondrial dysfunction and the perpetuation of inflammation.^[^ ^12^ ^]^ One approach involves the utilization of a camouflaged meta‐defensome, which scavenges mitochondrial ROS (mtROS) and inhibits mitochondrial nitric oxide synthase (mtNOS), ultimately facilitating a switch from mitochondrial glycolysis to oxidative phosphorylation. This metabolic reprogramming of mitochondrial activity promotes the repolarization of M1 synovial macrophages into the M2 phenotype.^[^ ^9^ ^]^ The polarization of M1 to M2 macrophages is highly dependent on the regulation of mitochondrial activity. Further exploration of these mechanisms holds significant potential for understanding and manipulating the balance of synovial macrophages in OA, paving the way for novel therapeutic interventions.

Astaxanthin (AST) is a naturally occurring fat‐soluble carotenoid renowned for its potent antioxidant, anti‐inflammatory, and immunomodulatory effects, making it a widely employed therapeutic agent in inflammation‐related diseases.^[^ ^13^ ^]^ With its structural composition characterized by a non‐polar region containing 13 conjugated diene unsaturated bonds, AST possesses exceptional antioxidant properties.^[^ ^13^, ^14^ ^]^ However, its lipophilic nature renders AST poorly soluble in aqueous solutions, significantly diminishing its bioavailability. Similarly, rapamycin (Rapa) serves as a potent activator of autophagy, thereby exerting anti‐inflammatory effects by inducing autophagic processes.^[^ ^15^ ^]^ A study by Lei et al. revealed that the delivery of Rapa into chondrocytes resulted in elevated levels of intracellular LC3‐II protein and reduced MMP13 production, confirming that Rapa promotes cellular autophagy and increases secretion of extracellular matrix in chondrocytes.^[^ ^16^ ^]^ However, the limited water solubility of Rapa hampers its administration efficiency via intra‐articular injection, thereby increasing the likelihood of exogenous cartilage damage and the risk of infection associated with repeated drug injections. Therefore, there is an urgent need to develop strategies to address the low effective utilization of small‐molecule AST and Rapa, while simultaneously avoiding the risk of drug accumulation at non‐inflammatory sites and reducing adverse side effects. Biodegradable nanomaterials have recently obtained much interest in arthritis inflammation.^[^ ^17^ ^]^ Numerous nanomaterials possessing certain physiological properties such as pH‐, ROS‐, and enzyme‐responsive have been developed in osteoarthritis.^[^ ^18^ ^]^ Concerned that a variety of ROS‐scavenging nanomaterials such as selenium/telluride‐containing polymers, diselenide/telluride‐containing polymers, and polyoxalates have been widely used for cancer treatment.^[^ ^19^ ^]^ However, these nanomaterials also have certain limitations such as difficulties in controllable synthesis.

**Scheme 1:**
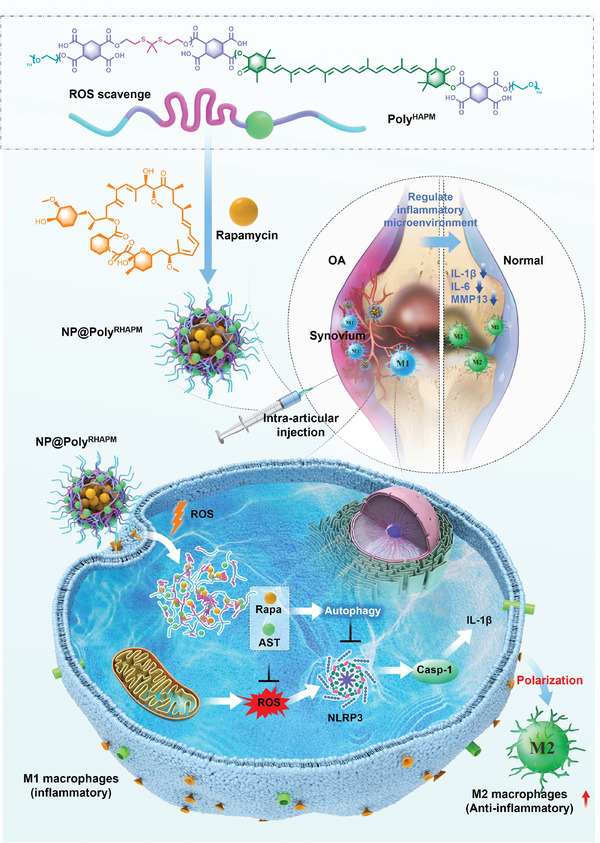
Schematic illustration of the effect of NP@Poly^RHAPM^ on polarizing macrophage in knee osteoarthritis. A ROS‐responsive biodegradable amphiphilic polymer (Poly^HAPM^) was designed, which was then applied to encapsulate rapamycin (Rapa) into the nanoparticle ( NP@Poly^RHAPM^). NP@Poly^RHAPM^ was then injected into the knee cavity. Once NP@Poly^RHAPM^ was taken up by synovial macrophages, on the one hand, the over‐expressed ROS of M1 macrophages could break up the thioketal bonds of the Poly^HAPM^, leading to ROS depletion. On the other hand, the free AST and Rapa could further scavenge ROS, resulting in the downregulation of NLRP3, cleaved caspase 1, and IL‐1β by enhancing autophagy. Moreover, NP@Poly^RHAPM^ successfully repolarized M1 macrophages to the M2 type, resulting in reducing the release of harmful inflammatory factors in the synovial environment, thereby protecting chondrocytes from damage.

In this study, we developed a ROS‐responsive nanocarrier system and tested the ROS‐scavenging ability and responsive release of AST/Rapa in vitro. Moreover, utilizing an anterior cruciate ligament transection (ACLT)‐induced OA mouse model to mimic chondrocyte injury in OA, intra‐articular injection of NP@Poly^RHAPM^ facilitated diffusion and macrophage uptake at the synovial inflammation site, resulting in articular cartilage protection and repolarization of synovial macrophages in vivo. The study revealed that excessive ROS levels in synovial M1 macrophages triggered the opening of the thioketal bond in NP@Poly^RHAPM^, leading to dissociation of the nanoparticles, subsequent release of AST, ROS consumption, and Rapa release, while free AST and Rapa effectively induced polarization of enhanced M1 macrophages toward the M2 phenotype by scavenging intracellular ROS, enhancing intracellular autophagy, removing damaged mitochondria, and inhibiting NLRP3 inflammatory vesicle activation (**Scheme** **1**), ultimately protecting chondrocytes from inflammatory damage. These findings demonstrate the promising potential of nanomedicine for the clinical treatment of OA.

## 2 Experimental Section

### 2.1 Materials

All chemicals were obtained from commercial sources and were used without further purification. Astaxanthin (AST), Rapamycin (Rapa), 1,2,4,5‐Cyclohexanetetracarboxylic Dianhydride (HPMDA), methoxypolyethylene glycols 5000 (mPEG_5k_), N, N‐dimethylformamide (DMF) and 3‐(4,5‐dimethylthiazol‐2‐yl)−2,5‐diphenyltetrazolium bromide (MTT) were purchased from Aladdin (Shanghai, China). Cell media, penicillin/streptomycin (P/S), 0.25% trypsin‐EDTA, and fetal bovine serum (FBS) were purchased from Gibco (Gran Island, NY, U.S.A.).

### 2.2 Synthesis of 2,2′‐(Propane‐2,2‐Diylbis(Sulfanediyl))Bis(Ethan‐1‐o1), PDSE

PDSE was synthesized and characterized as previously described in ref. [20].

### 2.3 Synthesis of Poly^HPP^

PDSE (0.1 mm) and 1,2,4,5‐Cyclohexanetetracarboxylic Dianhydride (HPMDA) (0.11 mm) were placed in a 50 mL round‐bottom flask, then 10 mL DMSO was added into the flask with continuous stirring for 48 h. Subsequently, mPEG_5k_‐OH (0.02 mmol) was added to endcap the polymer for 24 h. The final product poly (HPMDA‐co‐PDSE)‐PEG (Poly^HPP^) was collected via dialysis and then dried under a vacuum.^[^ ^21^ ^]^

### 2.4 Synthesis of Poly^HAPM^

Under nitrogen protection, astaxanthin (179 mg, 3 mmol) and PDSE (137 mg, 7 mmol) were dissolved in 20 mL of super‐dry DMF, stirred for 10 min at a temperature of 40 °C and protected from light, and then HPMDA (247 mg, 11 mmol) was dissolved in 10 mL of super‐dry DMF and stirred continuously for 48 h at 40 °C. mPEG_5k_‐OH (1 g, 2 mmol) was then added and stirred continuously at 40 °C for 24 h. Finally, the Poly^HAPM^ was obtained by dialysis and dried under vacuum, then analyzed by ^1^H NMR.

### 2.5 Preparation and Characterization of NP@Poly^RHPP^, NP@Poly^HAPM^ and NP@Poly^RHAPM^

Frist, the as‐synthesized Poly^HPP^ (10 mg) and Rapa (1 mg) were initially dissolved in 1 mL of DMSO, and then the solution was dispersed in 10 mL of de‐ionized water (43 °C) with continuous stirring. After vigorous stirring for 15 min, the mixture was collected and dialyzed using a dialysis bag (MWCO: 3500 Da) overnight. The nanoparticles (termed NP@Poly^RHPP^) were then separated by centrifugation and washed twice with de‐ionized water.

Then, a solution of Poly^HAPM^ (40 mg) in DMSO (1 mL) was added to deionized water (10 mL), dropwise with vigorous stirring. The mixture was dialyzed in a dialysis bag (MWCO: 3500 Da) for 12 h to obtain NP@Poly^HAPM^.

Finally, a solution of Rapamycin (4 mg) and Poly^HAPM^ (40 mg) in DMSO (1 mL) was added to deionized water (10 mL) dropwise under vigorously stirring. The mixture was dialyzed in a dialysis bag (MWCO: 3500 Da) for 12 h to obtain NP@Poly^RHAPM^.

The morphology of NP@Poly^HAPM^ and NP@Poly^RHAPM^ was characterized by TEM (HT‐7700, Hitachi, Japan). The size of NP@Poly^HAPM^ and NP@Poly^RHAPM^ was characterized by a Malvern Zetasizer Nano ZS90 laser particle size analyzer (Nano ZS, UK).

### 2.6 Stability of NP@Poly^HAPM^ and NP@Poly^RHAPM^

NP@Poly^HAPM^ was diluted in H_2_O solution and incubated for various time points (0, 1, 2, 3 days). Additionally, NP@Poly^RHAPM^ was incubated in a system of 10 mM H_2_O_2_ for 6 or 24 h. The size, polydispersity index (PDI), and zeta potential of NP@Poly^HAPM^ and NP@Poly^RHAPM^ were measured on a Malvern Zetasizer Nano ZS90 laser particle size analyzer, and the structure of NP@Poly^RHAPM^ was observed by TEM.

### 2.7 Drug release of NP@Poly^RHAPM^

NP@Poly^RHAPM^ was separately dissolved in PBS and hydrogen peroxide solution (10 mM) respectively. Then each solution was transferred into a pre‐swelled dialysis bag (MWCO: 3500 Da), which was then immersed into 100 mL of corresponding media in a shaking culture incubator at 37°C. At certain time points, 1 mL of sample solution was withdrawn from the dialysate and fresh solution (1 mL) was immediately added into the dialysate. All the samples were tested by HPLC. The Rapa released from the micelles was expressed as the percentage of cumulative Rapa in the dialysate to the total Rapa in the micelles.

### 2.8 In Vitro Antioxidant Activity

#### 2.8.1 H_2_O_2_ Scavenging Assay

The H_2_O_2_ scavenging activity of NP@Poly^RHAPM^ was evaluated with a Hydrogen peroxide test kit. First, NP@Poly^RHAPM^ (10 µm) was incubated in 2 ml of PBS containing 50 mM H_2_O_2_ at room temperature for 5 min, 1 h, 2 h, 4 h, 8 h, or various concentrations of NP@Poly^RHAPM^ (from 2.5, 5 to 10 µm) were incubated in 2 ml of PBS containing 50 mM H_2_O_2_ at room temperature for 2 h. Then added 50 µl of samples or standard solution (1, 2, 5, 10, 20, 50, and 100 µm) to a 96‐well plate. Next, 100 µL of hydrogen peroxide detection reagent was added to each well. After incubation at room temperature for 30 min, the concentration of remaining H_2_O_2_ was determined by measuring the absorption at 560 nm with a multiple plate reader, and the H_2_O_2_‐ scavenging capacity was calculated.

#### 2.8.2 ABTS^+^ Assay

The NP@Poly^RHAPM^ samples (10 µm) were mixed with the ABTS^+^ solution (7 mm) for 1 min, 30 min, 1 h, 2 h and 4 h in the dark. Subsequently, the absorbance of the mixture at 734 nm was measured with a plate reader. The ABTS^+^ scavenging activity of the NP@Poly^RHAPM^ was calculated based on the following equation: ABTS^+^ scavenging activity (%) = (A0 – Ai)/A0 × 100%. Where A0 and Ai represented the absorbance of ABTS^+^ solution before and after adding NP@Poly^RHAPM^ samples. The specific calculation method refers to the previous study.^[^ ^22^ ^]^

### 2.9 Cell Culturing and Treatment

**1)** RAW264.7 cells, a macrophage cell line that has the potential for differentiation, can induced to M1‐type macrophages modeling (M1 modeling) after 24 h induction by 100 ng mL^−1^ LPS plus 20 ng mL^−1^ IFN‐γ in vitro.^[^ ^23^ ^]^ RAW264.7 cells were cultured in DMEM medium supplemented with 10% fetal bovine serum and 1% penicillin−streptomycin solution; **2)** ATDC5 cells, the chondrocyte line that was induced to form chondrocytes by insulin‐transferrin‐selenium medium supplement (ITS). ATDC5 cells were cultured in DMEM‐F12 medium supplemented with 10% fetal bovine serum and 1% penicillin−streptomycin solution. These above cells were cultivated in a humidified atmosphere at 37°C with 5% of CO_2_.

### 2.10 Cellular Uptake Determined by Flow Cytometry

RAW264.7 cells were seeded at a density of 5 × 10^4^ cells/well in a 24‐well plate (Thermo Scientific, USA). After M1 modeling for 24 h, the media was removed and the cells were incubated with **NP@Poly^RHAPM^‐Cy5.5** diluted in cell media for various time intervals (1 h, 4 h, and 7 h). The cells were washed with phosphate‐buffered saline (PBS) for 3 times. The cellular uptake was assessed by flow cytometry. **NP@Poly^RHAPM^‐Cy5.5**: λ_ex_ = 673 nm, λ_em_ = 692 nm.

### 2.11 Cellular ROS Detection

RAW264.7 cells were seeded at a density of 1 × 10^5^ cells/well in a 24‐well plate, then cultured with LPS/IFN‐γ plus various treatment groups for 24 h. After that, DCFH‐DA was diluted 1:1000 in serum‐free medium to a final concentration of 10 µm, which was incubated at 37°C with cells for 0.5 h in the dark. Next, cells were washed three times to sufficiently remove DCFH‐DA that did not enter the cells. After counterstaining with Hoechst, the fluorescent images were observed in CLSM. DCFH‐DA: λ_ex_ = 488 nm, λ_em_ = 525 nm. Hoechst: λ_ex_ = 405 nm, λ_em_ = 460 nm. Meanwhile, the mean fluorescence intensity of cellular ROS was detected by FCM, with an excitation wavelength of 488 nm and an emission wavelength of 520 nm. The data were quantified and then presented by FlowJo software (Tree Star, OR, USA).

### 2.12 Cell Viability Assay

#### 2.12.1 Live/Dead Stain

RAW264.7 cells were seeded at a density of 5 × 10^5^ cells/well in 6‐well plates (Thermo Scientific, USA), then cultured with LPS/IFN‐γ plus various concentrations NP@Poly^RHAPM^ (0, 1.25, 2.5, 5, 10 and 20 µM) for 24 h. After that, the media was removed, and the cells were washed with PBS. The cells were further incubated with Calcein‐AM/propidium iodide (1:1000, KeyGEN BioTECH) for 15 min. The cells were washed with PBS and the cell survival/death was assessed by CLSM. Calcein‐AM: λ_ex_ = 495 nm, λ_em_ = 515 nm, propidium iodide: λ_ex_ = 488 nm, λ_em_ = 630 nm.

#### 2.12.2 MTT Assay

The cytotoxicity of the cells was assessed using a (3‐(4,5‐dimethylthiazol‐2‐yl)−2,5‐diphenyltetrazolium bromide) (MTT) colorimetric assay. Briefly, RAW264.7 cells were seeded at a density of 5000 cells/well in a 96‐well plate. After 24 h, the media was removed, and the cells were incubated with increasing concentrations of NP@Poly^RHAPM^ diluted in cell media for 24 and 48 h. After this time, the media was replaced with fresh media containing MTT (10 µL of a 5 mg mL^−1^ solution in PBS), and the cells were further incubated for 4 h. Acidified SDS solution was then added (100 µL/well) and the plates kept in the dark for an additional 12 h. Absorption measurements were performed on a Bio‐Rad plate reader at 570 nm (peak absorbance) and at 650 nm (background absorbance).

### 2.13 Western Blotting

The expressions of COX‐2, LC3‐II/I, p62, NLRP3, ASC, Cleaved caspase‐1, IL‐1β and MMP13 proteins were detected by Western blotting. Cell lysate (RIPA: protease inhibitor: phosphorylated protease inhibitor = 100:1:1) was added to the cell dish to extract all proteins in the cell. The protein was quantified by the Coomassie brilliant blue protein quantitation method. An appropriate separation gel was selected according to the molecular weight. 30 µg of protein sample was added per lane, and the samples were separated by SDS‐PAGE gel electrophoresis. Then the protein was transferred to the polyvinylidene fluoride (PVDF) membrane. 5% milk or 5% BSA was used for blocking for one hour. The membranes were then incubated with the primary antibodies (β‐actin, COX‐2, LC3‐II/I, p62, NLRP3, ASC, Cleaved caspase‐1, IL‐1β and MMP13, 1:1000) overnight on a shaker at 4°C. Subsequently, the fluorescent secondary antibodies were incubated for 2 h at room temperature and the Western blot images were visualized by a Gel imaging system (Tanon 4800, China).

### 2.14 Macrophage Polarization Assessment

RAW264.7 cells were seeded at a density of 5 × 10^4^ cells/well in a 24‐well plate, then cultured with LPS/IFN‐γ plus various treatment groups for 24 h. After that, the cells were fixed in 4% paraformaldehyde with 0.1% Triton X‐100 (Sigma–Aldrich). Furthermore, the cells were treated with 1% BSA to block nonspecific binding and stained with anti‐CD80 and anti‐CD206 antibody (BD, CA, USA; dilution, 1:200). The cells were stained with fluorescent secondary antibodies and the nucleus‐specific stain DAPI. Then the images were observed via CLSM. Image J software was used for semi‐quantitative analysis. Meanwhile, flow cytometry was used for quantitative detection of mean fluorescence intensity. The data were quantified and then presented by FlowJo software.

### 2.15 Macrophages Conditioned Medium (CM) Collection

RAW264.7 cells were seeded at a density of 1 × 10^6^ cells/well in a 6‐well plate, then incubated with LPS/IFN‐γ plus various treatment groups for 24 h. After that, the supernatants of polarized macrophages were collected for centrifuging at 1000 g for 5 min and then stored at −80°C for further experiments. The macrophage CM was diluted with an equal volume of serum‐free DMEM‐F12 medium and added to the culture dish for culturing chondrocytes.

### 2.16 ELISA Assay

RAW264.7 cells were seeded at a density of 3 × 10^5^ cells/well in a 12‐well plate overnight, then incubated with LPS/IFN‐γ plus various treatment groups for 24 h. After that, the cells were centrifuged at 12,000 rpm min^−1^ for 5 min. The obtained supernatant using a mouse IL‐1β high sensitivity ELISA kit (EK201BHSS, Multi Sciences) and a mouse IL‐6 ELISA kit (EK2236‐01, Multi Sciences) according to the manufacturer protocol.

### 2.17 Apoptosis Rate of ATDC5 Cells

The cell apoptosis rate was detected using an annexin V‐FITC apoptosis detection Kit (Elabscience). Briefly, ATDC5 cells were seeded at a density of 1 × 10^6^ cells/well in 6‐well plates overnight, then the cells were incubated with CM of various treatment groups for 48 h. After that, the dead and live cells were collected for centrifuging at 1000 g for 5 min and further incubated with Annexin V/PI (1:100 dye solution) for 15 min. The cells were washed with PBS and the cell death was assessed by FCM. Annexin V: λ_ex_ = 488 nm, λ_em_ = 530 nm, PI: λ_ex_ = 488 nm, λ_em_ = 630 nm. The data were quantified and then presented by FlowJo software.

### 2.18 IVIS Imaging

NP@Poly^RHAPM^ were labeled with Cy7.5 (designated as NP@Poly^RHAPM^‐Cy7.5) to monitor their localization *in vivo via* an in vivo imaging system (IVIS, Lumina Series III, PerkinElmer, USA). NP@Poly^RHAPM^‐Cy7.5 was injected into the knee joint and their biofluorescence intensity in the knee joint was observed at different time points. To investigate the metabolic pathway of NP@Poly^RHAPM^‐Cy7.5, it was injected into the knee joint and it's biodistribution in each organ was observed after 48 h.

### 2.19 OA Model Establishment

Anterior Cruciate Ligament Transection (ACLT) was administrated for constructing the OA model, as described in previous research.^[^ ^24^ ^]^ Thirty C57BL/6 mice (8‐week‐old, male) were acquired from Vital River Laboratory Animal Technology Co. Ltd. (Beijing, China). An anterior drawer test was performed to confirm the complete transection of anterior cruciate ligament . The mice were randomly split into 5 groups. Mice in group 1 were treated with sham surgery, which opened only the joint capsule and then sutured the incision. The right knee underwent ACLT in mice from groups 2–5. Specifically, mice in group 2 received an intra‐articular injection of saline, while mice in groups 3–5 received an intra‐articular injection of 20 µL of various treatment groups (Rapa, NP@Poly^HAPM,^ or NP@Poly^RHAPM^) every 3 days for 6 weeks.

### 2.20 Histological and Immunohistochemical Analysis

After 6 weeks of injection, the mice were sacrificed, and the right knee joints were harvested and fixed in 4% paraformaldehyde. Further, the samples were decalcified in 10% EDTA (pH 7.4) for 4 weeks and then embedded in paraffin. Sagittal sections of the medial compartment of the knee joint were cut at 4 µm. Histological sections were stained with H&E and safranin O‐fast green (S&F). Immunohistochemical (IHC) staining was performed with antibody against CD80 and CD206 (BD, CA, USA; dilution 1:200). The percentage of positively stained cells in the synovium and articular cartilage was quantified by Image J software.

### 2.21 Statistical Analysis

GraphPad prism 8.0 was used for data analysis. Data were presented as mean ± standard deviation (SD). The student's t‐test was used for the determination of statistical significance between the 2 groups. The ANOVA test was used for the determination of statistical significance between multiple groups. Differences were considered statistically significant at a level of ^*^ *p*<0.05, ^**^ *p*<0.01, &*p*<0.001 and ^#^ *p*<0.0001.

## 3 Results and Discussion

### 3.1 Synthesis and Characterization of NP@Poly^RHAPM^

**Figure 1:**
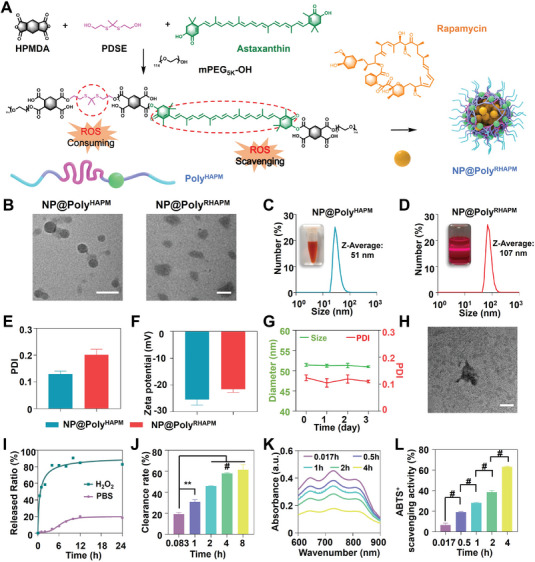
Characterization of NP@Poly^HAPM^ and NP@Poly^RHAPM^. A) Schematic illustration of the synthesis of biodegradable Poly^HAPM^ via condensation polymerization with astaxanthin (AST), HPMDA, and PDSE, which was then end‐capped with mPEG_5k_‐OH. Subsequently, Rapa was encapsulated with Poly^HAPM^ to give NP@Poly^RHAPM^. The thioketal bond of Poly^HAPM^ can be broken by ROS, thereby consuming ROS, and AST conjugated double bond can be oxidized by ROS, thereby depleting ROS. B) TEM of NP@Poly^HAPM^ and NP@Poly^RHAPM^. Scale bar: 100 nm. C,D) Average size, E) PDI, and F) Zeta potentials of NP@Poly^HAPM^ and NP@Poly^RHAPM^ were determined by DLS. G) The average size and PDI of NP@Poly^HAPM^ in H_2_O solution at various times were determined by DLS. H) TEM image of NP@Poly^RHAPM^ at H_2_O_2_ solution at 24 h. Scale bar: 250 nm. I) Release ratio of Rapa in PBS and H_2_O_2_ solutions at different time points. J) Scavenging H_2_O_2_ by NP@Poly^RHAPM^ at various time points. K,L) The scavenging ability of ABTS^+^ by NP@Poly^RHAPM^ at different time points. Data are presented as the mean ± SD (*n = 3*). Statistical significance between every two groups was calculated via a one‐way ANOVA test. ^**^ *p*<0.01 and ^#^ *p*<0.0001.

Astaxanthin (AST), 2,2′‐(propane‐2,2‐diylbis(sulfanediyl))bis(ethan‐1‐ol) (PDSE) and 1,2,4,5‐Cyclohexanetetracarboxylic Dianhydride (HPMDA) were polymerized through condensation polymerization, followed by end‐capping with a hydrophilic polymer mPEG_5k_, resulting in the synthesis of a ROS‐responsive biodegradable amphiphilic polymer (i.e., Poly^HAPM^). ^1^H NMR was used to confirm the effective production of Poly^HAPM^ (Figure S1, Supporting Information). Subsequently, the Poly^HAPM^ was used to synthesize nanoparticles (i.e., NP@Poly^HAPM^) through dissolution and dialysis. Moreover, Rapa was physically wrapped utilizing NP@Poly^HAPM^ using the nanoprecipitation process to generate Rapa‐loaded nanoparticles NP@Poly^RHAPM^ (**Figure** **1**A). Given that AST has a distinct absorption region in the ultraviolet–visible light region (Figure S2, Supporting Information), the AST content of NP@Poly^RHAPM^ was determined using a plate reader, and the Rapa content of one was determined using high‐performance liquid chromatography (HPLC). The results indicated an approximate AST to Rapa content ratio of 1:1.

To study the physic‐chemical properties of the above NPs (e.g., NP@Poly^HAPM^ and NP@Poly^RHAPM^), transmission electron microscopy (TEM) was employed, and the results showed that the particle sizes of NP@Poly^HAPM^ and NP@Poly^RHAPM^ were uniformly complete spheroids with size of ≈50 and 100 nm, respectively (Figure 1B). Dynamic light scattering (DLS) analysis revealed that the average hydrodynamic diameter of NP@Poly^HAPM^ was ≈51 nm (Figure 1C), whereas the size of NP@Poly^RHAPM^ was 107 nm (Figure 1D). The polydispersity index (PDI) of NP@Poly^HAPM^ was ≈0.13 and the Zeta potential was ≈−25 mV. those of NP@Poly^RHAPM^ were 0.20 and −21 mV, respectively (Figure 1E,F). Additionally, the average particle size and PDI changes of NP@Poly^HAPM^ at various times were observed by DLS. As seen in Figure 1G, no significant changes were observed in the average particle size and PDI of NP@Poly^HAPM^ in H_2_O at different times (0 to 3 days), which proved the superior stability of NP@Poly^HAPM^. Collectively, those results suggest both NP@Poly^HAPM^ and NP@Poly^RHAPM^ are stable and well‐dispersed subspherical nanoparticles.

Furthermore, after NP@Poly^RHAPM^ entered the cell, the high‐level ROS could break the thioketal bond in the polymer main chain, leading NP@Poly^RHAPM^ to dissociate and release the loaded Rapa. The dissociation and drug release behavior of NP@Poly^RHAPM^ in the presence of H_2_O_2_ were investigated. DLS characterization revealed that when NP@Poly^RHAPM^ was treated with 10 mM H_2_O_2_ for 6 and 24 h, the average particle size increased from 122.3 to 507.0 nm and the PDI increased from 0.205 to 0.539 (Figure S3, Supporting Information). Furthermore, the Zeta potential of NP@Poly^RHAPM^ was slightly increased after treating with H_2_O_2_ (Figure S4, Supporting Information). The TEM images provided further confirmation that, following 24 h of H_2_O_2_ treatment, NP@Poly^RHAPM^ experienced substantial dissociation. Moreover, the previously homogeneous spherical structure of NP@Poly^RHAPM^ transitioned into an irregular morphology (Figure 1H). In summary, these results demonstrate the ROS‐responsive nature of NP@Poly^RHAPM^.

Subsequently, the release behavior of Rapa wrapped in NP@Poly^RHAPM^ was detected by using HPLC in different solutions. The outcomes shown that the release of wrapped Rapa reached ≈82.8% when NP@Poly^RHAPM^ was added to a 10 mm H_2_O_2_ environment at 24 h. Only 18.5% of Rapa wrapped in NP@Poly^RHAPM^ in PBS solution, however, was released within the same time frame (Figure 1I). The fact that NP@Poly^RHAPM^ does indeed exhibit ROS‐sensitive responsive release properties is once again highlighted by this. Moreover, to determine whether AST was successfully polymerized on the main chain of polymer NP@Poly^HAPM^, the ultraviolet enzyme labeler was used. The shared characteristic peak of AST, NP@Poly^HAPM,^ and NP@Poly^RHAPM^ at 490 nm indicated the successful self‐assembly of AST within NP@Poly^HAPM^ (Figure S5, Supporting Information).

As ROS can be eliminated by the thioketal bond of NP@Poly^RHAPM^ and AST on the chain, H_2_O_2_ and 2, 2′‐azino‐bis (3‐ethylbenzothiazoline‐6‐sulfonic acid) diammonium salt‐free radicals (ABTS^+^) were chosen to test the capacity of NP@Poly^RHAPM^ to remove ROS.^[^ ^22^ ^]^ The results demonstrated that the removal of H_2_O_2_ by NP@Poly^RHAPM^ was time‐dependent. After 8 h of co‐incubation, NP@Poly^RHAPM^ removed 61.5% of H_2_O_2_ (Figure 1J), and this removal ability was dose‐dependent (Figure S6, Supporting Information). NP@Poly^HAPM^ exhibited a consistent trend in its ability to scavenge H_2_O_2_ (Figure S7, Supporting Information). Similarly, we assessed the capacity of NP@Poly^RHAPM^ to remove ABTS^+^. The results indicated that the clearance of ABTS^+^ by NP@Poly^RHAPM^ was both concentration‐ and time‐dependent. The clearance rate of ABTS^+^ increased proportionally with the concentration of NP@Poly^RHAPM^ (Figure S8, Supporting Information). After 4 h of co‐incubation, the clearance rate of ABTS^+^ reached ≈63.3% (Figure 1K,L). The ability of NP@Poly^RHAPM^ to effectively remove ROS provides a theoretical basis for protecting cartilage cells from the detrimental effects of oxidative stress.

### 3.2 Cellular Uptake and ROS Scavenging Ability of NP@Poly^RHAPM^ In Vitro

**Figure 2:**
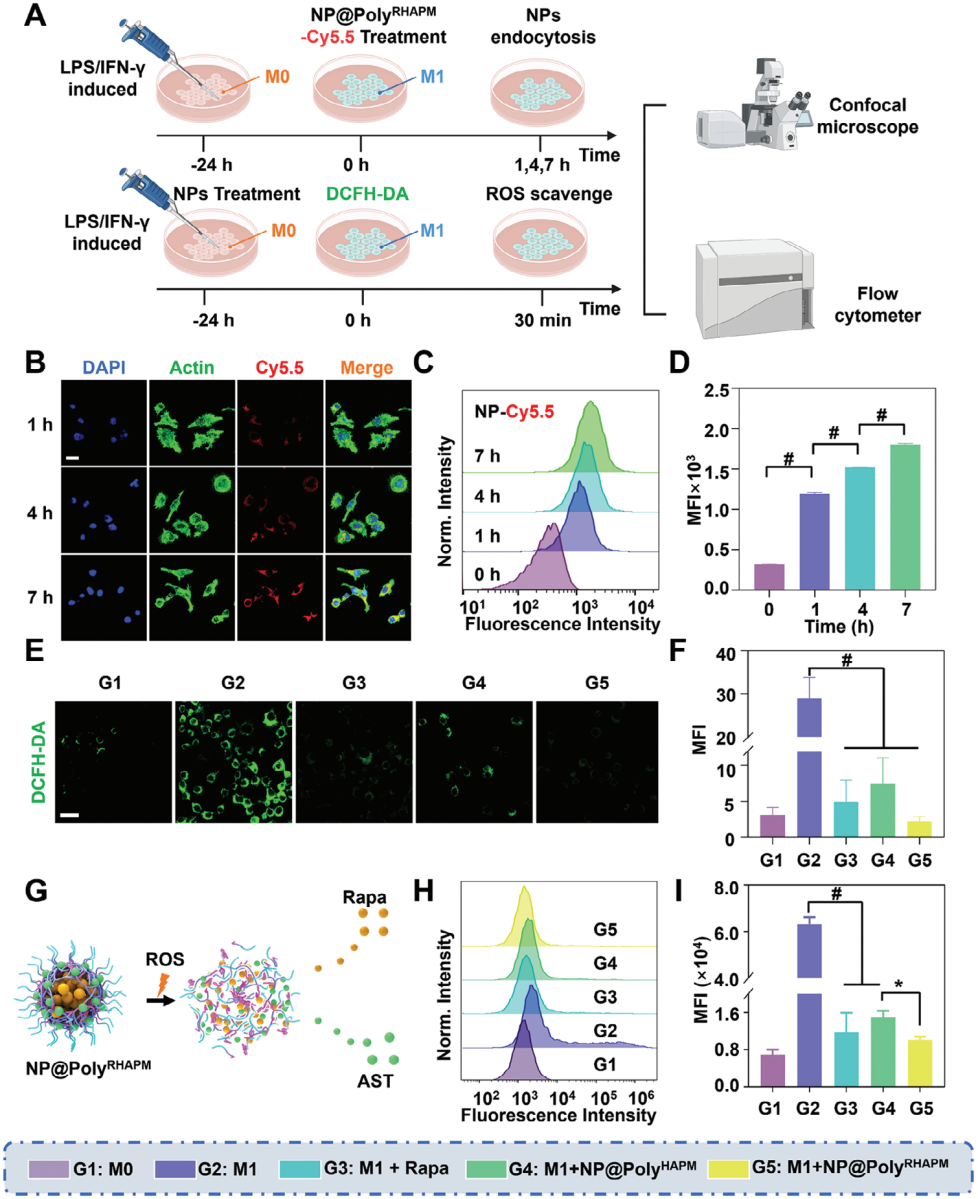
Cellular uptake and ROS scavenging ability of NP@Poly^RHAPM^ in vitro. A) Schematic diagram of cellular uptake and ROS scavenging. B) CLSM images of RAW264.7 cells treated with NP@Cy5.5 at 1, 4, and 7 h. Nuclei were filtered with blue fluorescence by DAPI. Red fluorescence and green fluorescence were obtained from Cy5.5 and Alexa‐488 actin‐stained cytoskeleton, respectively. Scale bar: 20 µm. C,D) Quantitative analysis of cellular uptake of NP@Poly^RHAPM^ at different time points using FCM. E,F) CLSM images of macrophages cultured with the ROS probe DCFH‐DA (green) to directly observe intracellular ROS levels and to perform a semi‐quantitative analysis of ROS levels. Scale bar: 20 µm. G) Schematic diagram of Rapa release by NP@Poly^RHAPM^ in the presence of ROS. H,I) Quantitative analysis of intracellular ROS levels in macrophages using FCM. Data are presented as the mean ± SD (*n = 3*). Statistical significance between every 2 groups was calculated via a one‐way ANOVA test. ^*^ *p*<0.05 and ^#^ *p*<0.0001.

Laser confocal microscopy (CLSM) and flow cytometry (FCM) were used to assess the in vitro cellular absorption and ROS scavenging capacity of NP@Poly^RHAPM^ (**Figure** **2**A). M1 macrophages were inducted by lipopolysaccharide (LPS) and interferon‐γ (IFN‐γ) of RAW264.7 cells (referred to as M0 macrophages), as shown by FCM. NP@Poly^RHAPM^ was marked with the red fluorescent dye Cy5.5, defined as NP‐Cy5.5. The results revealed that M1 macrophages exhibited significant red fluorescence primarily localized in the cytoplasm, indicating effective internalization of NP@Poly^RHAPM^ by the macrophages (Figure 2B). Furthermore, the red fluorescence intensity of M1 macrophages at 7 h was 1.19 times higher than that at 4 h and 1.51 times higher than that at 1 h, based on quantitative measurement of intracellular Cy5.5 fluorescence intensity using FCM (Figure 2C,D). These findings collectively showed that NP@Poly^RHAPM^ could efficiently enter M1 macrophages in a time‐dependent manner.

The intracellular ROS levels of macrophages were then detected using the reactive oxygen probe DCFH‐DA after various treatments to further assess the ROS clearance capacity of NP@Poly^RHAPM^. DCFH‐DA is a ROS‐specific probe that can be oxidized by ROS in cells to produce DCF, which emits strong green fluorescence. The degree of ROS in the cell is inversely correlated with the strength of the green fluorescence. The results demonstrated that M1 macrophages displayed high green fluorescence, indicating that their intracellular ROS levels were greatly raised when the lowest intracellular ROS level of M0 macrophages was employed as a reference. However, the strongest drop in intracellular green fluorescence intensity was observed in M1 macrophages treated with NP@Poly^RHAPM^, showing that its ROS elimination ability was far superior to that of any other treatment group. Rapa and NP@Poly^HAPM^ both could eliminate ROS, as shown by the fact that M1 macrophages treated with them both had considerably dimmer green fluorescence than the M1 group (Figure 2E). Additionally, Image‐J software was used to do a semi‐quantitative analysis of the intracellular ROS levels of macrophages. According to the findings, intracellular ROS levels were 13.0 times lower in M1 macrophages treated with NP@Poly^RHAPM^ than in the M1 group (Figure 2F). Based on the excellent ROS response and clearance ability of NP@Poly^HAPM^, Rapa released by NP@Poly^RHAPM^ can further improve its intracellular ROS clearance capacity (Figure 2G). To further quantify the intracellular levels of ROS, the intracellular ROS levels of macrophages were assessed using FCM (Figure 2H). The results revealed that the intracellular ROS level in M1 macrophages treated with NP@Poly^RHAPM^ was only ≈15.9% of that in the M1 group (Figure 2I). Additionally, the capacity of NP@Poly^HAPM^ and NP@Poly^RHAPM^ to remove ROS is concentration‐dependent (Figure S9, Supporting Information). Accordingly, under the stimulation of LPS plus IFN‐γ, RAW264.7 cells were polarized to form M1 macrophages with noticeably increased intracellular ROS levels. Following treatment with Rapa, NP@Poly^HAPM^, and NP@Poly^RHAPM^, the intracellular ROS level of M1 macrophages was significantly reduced. Notably, NP@Poly^RHAPM^ exhibited the lowest ROS levels, indicating its antioxidative activity.

### 3.3 Anti‐Inflammatory Mechanism of NP@Poly^RHAPM^ in Macrophages

**Figure 3:**
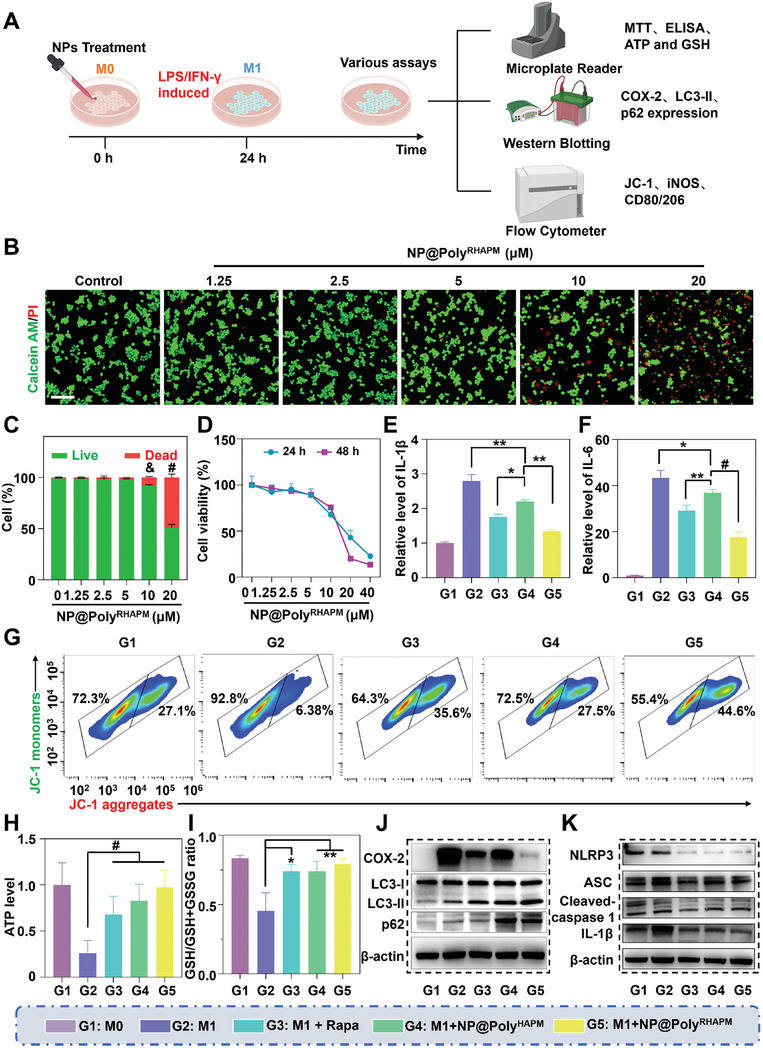
Anti‐inflammatory mechanism of NP@Poly^RHAPM^ in macrophages. A) Schematic diagram to explore the anti‐inflammatory mechanism of NP@Poly^RHAPM^. B) Live/dead staining of macrophages treated with various concentrations of NP@Poly^RHAPM^ for 24 h. Scale bar: 100 µm. C) Quantitative analysis of Calcein‐AM and PI‐positive cells treated with various concentrations of NP@Poly^RHAPM^ for 24 h. D) MTT assay of macrophages treated with various concentrations of NP@Poly^RHAPM^ for 24 and 48 h. E,F) The level of IL‐1β and IL‐6 in M1 macrophages treated with different drugs was determined using ELISA. G) Mitochondrial membrane potential within M1 macrophages after different drug treatments was detected by flow cytometry. H) Bar graph of relative ATP production in M1 macrophages after NP@Poly^RHAPM^ treatment. I) Values of GSH/(GSH + GSSG) in M1 macrophages after various treatments. J) Western blot showing the expression of COX‐2, LC3‐II/I, and p62 in macrophages after various treatments. K) Western blot showing the expression of NLRP3, ASC, cleaved‐caspase 1, and IL‐1β in macrophages after various treatments. Data are presented as the mean ± SD (*n = 3*). Statistical significance between every 2 groups was calculated via a one‐way ANOVA test. ^*^ *p*<0.05, ^**^ *p*<0.01, &*p*<0.001 and ^#^ *p*<0.0001.

To elucidate the mechanism by which NP@Poly^RHAPM^ attenuates the inflammatory response in M1 macrophages, co‐incubation experiments were conducted using various treatments for 24 h (**Figure** **3**A). First, a live/dead staining was used to evaluate the impact of NP@Poly^RHAPM^ on M0 macrophages at various doses. As shown in Figure 3B,C, the results demonstrated that after 24 h of treatment, there was essentially no impact on the viability of RAW264.7 cells when the concentration of NP@Poly^RHAPM^ was 5 µm or below. However, at concentrations of 10 and 20 µm, the semi‐quantitative examination of the live/dead staining revealed an increase in PI‐positive cells and a decrease in Calcein‐AM‐positive cells. To further study the effect of NP@Poly^RHAPM^ on cell viability, the toxic effects of NP@Poly^RHAPM^ were determined using the MTT assay on M0 macrophages at 24 and 48 h. The results showed that the cytotoxicity of NP@Poly^RHAPM^ on RAW264.7 cells at a dose of 5 µm was negligible. When the concentration of NP@Poly^RHAPM^ reached 10  µm or higher, cell viability decreased in a dose‐dependent manner (Figure 3D). Similar cytotoxicity results were observed after treatment with NP@Poly^HAPM^ (Figure S10, Supporting Information). As a result, the starting concentration for NP@Poly^HAPM^ and NP@Poly^RHAPM^ in the following experiment was set at 5 µm.

IL‐1β and IL‐6 are crucial inflammatory mediators released by M1 macrophages and are involved in the degeneration of joint cartilage.^[^ ^25^ ^]^ To investigate whether NP@Poly^RHAPM^ might restrict the release of inflammatory mediators in M1 macrophages, the levels of IL‐1β and IL‐6 in cell supernatants were measured using an enzyme‐linked immunosorbent assay (ELISA). The results demonstrated that IL‐1β and IL‐6 were significantly generated by M1 macrophages. Rapa, NP@Poly^HAPM^, and NP@Poly^RHAPM^ treatment, however, significantly reduced IL‐1β and IL‐6 secretion in M1 macrophages. It is significant to notice that IL‐1β and IL‐6 secretion were the ones that were most severely suppressed by NP@Poly^RHAPM^ (Figure 3E,F; Figure S11, Supporting Information). These results imply that NP@Poly^RHAPM^ can inhibit the release of inflammatory mediators, hence protecting cartilage cells subtly.

Studies have indicated that elevated levels of iNOS and H_2_O_2_ in M1 macrophages disrupt mitochondrial energy metabolism, which enhances the resistance to oxidative damage and promotes the secretion of inflammatory factors.^[^ ^11^, ^26^ ^]^ The change in mitochondrial membrane potential in macrophages was assessed using FCM after various treatments to determine whether NP@Poly^RHAPM^ had a protective impact on the integrity of mitochondrial membrane potential. M1 macrophages had a lower ratio of JC‐1 aggregates/monomers than M0 macrophages, which indicated that their mitochondrial membrane potential had drastically diminished. This ratio increased dramatically in M1 macrophages after treatment with Rapa, NP@Poly^HAPM,^ and NP@Poly^RHAPM^, proving that NP@Poly^RHAPM^ was successful in restoring the mitochondrial membrane potential in M1 macrophages (Figure 3G; Figure S12, Supporting Information). Additionally, the regulatory effect of NP@Poly^RHAPM^ on energy metabolism was evaluated in M1 macrophages using the adenosine triphosphatase (ATP) assay. As showed in Figure 3H, NP@Poly^RHAPM^ greatly boosted intracellular ATP synthesis, demonstrating that it could restore mitochondrial energy metabolism in macrophages.

Additionally, the ratio of intracellular GSH to GSSG can be used as a measure of an organism's ability to withstand oxidative stress. Intracellular GSH is a protective antioxidant that is consumed excessively in an inflammatory environment.^[^ ^27^ ^]^ A GSH assay kit was used to determine the amount of GSH present in macrophages. Rapa, NP@Poly^HAPM^, and NP@Poly^RHAPM^ all considerably improved the intracellular GSH/ (GSH + GSSG) ratio, providing further evidence of the ability of NP@Poly^RHAPM^ to suppress macrophage responsiveness to oxidative stress (Figure 3I).

The cyclooxygenase‐2 (COX‐2) expression in macrophages after treated with NP@Poly^RHAPM^ was investigated utilizing western blotting to explore the anti‐inflammatory molecular mechanism. The COX‐2 relative protein expression in M1 macrophages was significantly higher than in the M0 group. Rapa, NP@Poly^HAPM^, and NP@Poly^RHAPM^ dramatically reduced the relative protein expression of COX‐2 in M1 macrophages. Notably, NP@Poly^RHAPM^ exhibited the most pronounced downregulation of COX‐2 protein expression in M1 macrophages (Figure 3J; Figure S13, Supporting Information).

By removing free radicals, the metabolic decomposition process known as autophagy can improve cellular microcirculation. According to reports, mitochondrial autophagy is also significantly influenced by the autophagy marker LC3‐II. Its purpose is to provide for its own energy requirements and control the quality of the mitochondria by removing damaged mitochondria and reducing excessive ROS production. After being treated with Rapa, NP@Poly^HAPM^, and NP@Poly^RHAPM^, the results demonstrated that, in comparison to the M1 group, the intracellular LC3‐II/I ratio of M1 macrophages increased greatly and the relative protein expression of p62 increased significantly (Figure 3J; Figure S14 and S15, Supporting Information).

Additionally, it was reported that autophagy also limits the activation of NLRP3 inflammatory vesicles and subsequently prevents persistent inflammation.^[^ ^28^ ^]^ To this end, the relative protein expression of NLRP3 in macrophages was further measured by using western blotting. The result shown that NP@Poly^RHAPM^ significantly reduced the relative protein expression of NLRP3, ASC, cleaved‐caspase 1, and IL‐1β in M1 macrophages (Figure 3K; Figure S16, Supporting Information). Overall, these results suggest that NP@Poly^RHAPM^ enhances the level of autophagy in macrophages to regulate intracellular mitochondrial mass and inhibit the activation of NLRP3 inflammatory vesicles.

### 3.4 NP@Poly^RHAPM^ Regulates Macrophage Polarization

**Figure 4:**
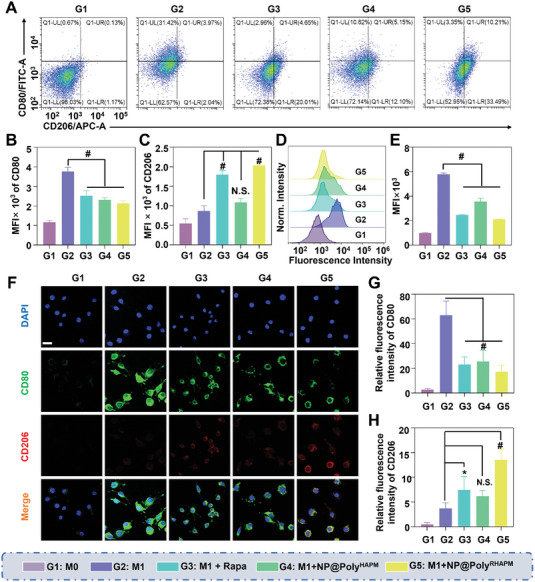
Effect of NP@Poly^RHAPM^ on macrophage repolarization. A) Relative fluorescence amounts of M1 (CD80^+^, CD206^−^) and M2 (CD206^+^, CD80^−^) macrophages were assessed by FCM, and B,C) the data were quantified. D,E) The expression of iNOS was measured and quantified using FCM. F) CLSM was used to detect the effects of different drug treatments on relative fluorescence amounts in macrophages and G,H) semiquantitative analysis of the relative fluorescence amounts of CD80 and CD206. Scale bar: 50 µm. Data are presented as the mean ± SD (*n = 3*). Statistical significance between every 2 groups was calculated via a one‐way ANOVA test. N.S. means no significance, ^*^ *p*<0.05, ^**^ *p*<0.01, &*p*<0.001 and ^#^ *p*<0.0001.

Growing evidence suggests a strong correlation between the severity of OA and the imbalance of synovial M1/M2 macrophages.^[^ ^9^, ^23^ ^]^ Repolarizing M1 macrophages into M2 macrophages has been shown to successfully halt the development of OA.^[^ ^29^ ^]^ By designating CD80^+^ as an M1 marker and CD206^+^ as an M2 marker, the average fluorescence intensity in macrophages was detected using FCM to assess the impact of NP@Poly^RHAPM^ on macrophage repolarization. The findings demonstrated that the average fluorescence intensity of CD80^+^ in M1 macrophages was significantly higher than that of the M0 group, and that of CD206^+^ was also elevated to some extent. However, following treatment with Rapa, NP@Poly^HAPM^, and NP@Poly^RHAPM^, the average fluorescence intensity of CD80^+^ in M1 macrophages was significantly decreased and the average fluorescence intensity of CD206^+^ increased significantly, though the increase in the average fluorescence intensity of CD206^+^ in the NP@Poly^HAPM^ treatment group was not significant (**Figure** **4**A). In comparison to the M1 group, NP@Poly^RHAPM^ decreased the average fluorescence intensity of CD80^+^ in M1 macrophages by 43.5% and increased the average fluorescence intensity of CD206^+^ by 234.14% (Figure 4B,C), showing that NP@Poly^RHAPM^ could significantly inhibit M1 macrophage polarization and promote M2 macrophage repolarization.

iNOS, which is highly expressed by M1 macrophages, is frequently used to identify them.^[^ ^9^ ^]^ To this goal, FCM was used to gauge the average fluorescence intensity of iNOS. The findings demonstrated that the average fluorescence intensity of iNOS in M1 macrophages was substantially higher than that of the M0 group. Rapa, NP@Poly^HAPM,^ and NP@Poly^RHAPM^ therapy, however, dramatically decreased the average fluorescence intensity of iNOS in M1 macrophages (Figure 4D,E), demonstrating once more that NP@Poly^RHAPM^ could effectively prevent M1 macrophage polarization. Moreover, the outcomes of CD80^+^ and CD206^+^ immunofluorescence staining agreed with those of FCM. NP@Poly^RHAPM^ considerably reduced the green fluorescence intensity of M1 macrophages while greatly increasing the red fluorescence intensity, according to immunofluorescence data (Figure 4F). Further semi‐quantitative analysis of these data revealed that, when compared to the M1 group, NP@Poly^RHAPM^ increased CD206^+^ relative fluorescence intensity by 3.6 times and decreased CD80^+^ relative fluorescence intensity by 3.7 times in M1 macrophages (Figure 4G,H). NP@Poly^RHAPM^ was able to repolarize M1 macrophages into M2 type, which decreased the release of detrimental inflammatory components in the synovial environment, according to the combined results of all these studies.

### 3.5 Anti‐Inflammatory Mechanism of NP@Poly^R^ ^HAPM^ Evaluated by RNA Sequencing Analysis

**Figure 5:**
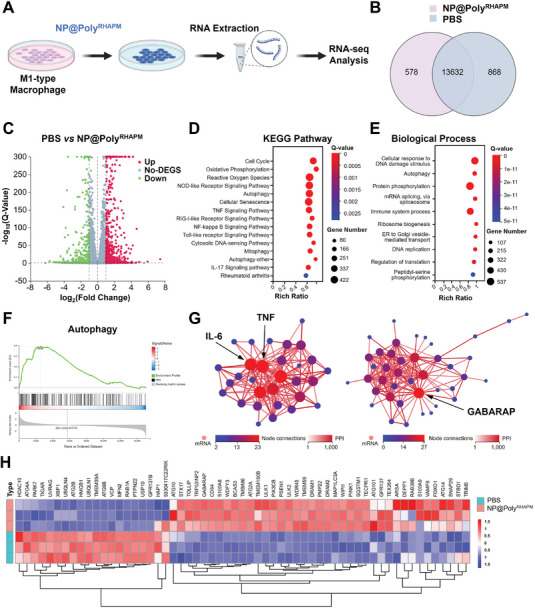
RNA‐seq analysis of the anti‐inflammatory mechanisms of NP@Poly^RHAPM^. A) Schematic diagram of M1‐type macrophages treated with NP@Poly^RHAPM^ by RNA‐Seq analysis. B) Venn diagram of gene expression in M1‐type cells treated with PBS and NP@Poly^RHAPM^. C) Volcano plot showing the genes regulated by the treatment of NP@Poly^RHAPM^. D) KEGG pathway enrichment analysis of differentially expressed genes in PBS group and NP@Poly^RHAPM^ treatment group. E) Bubble diagram of biological processes of differentially expressed genes in PBS group and NP@Poly^RHAPM^ treatment group. F) GSEA analysis showing differentially expressed genes in autophagy in PBS group and NP@Poly^RHAPM^ treatment group. G) PPI analysis of the differentially expressed core genes in positive regulation of interleukin‐1 beta production and autophagy. H) Heatmap analysis of core genes on autophagy in PBS group and NP@Poly^RHAPM^ treatment group.

To investigate further insights into the anti‐inflammatory mechanism of NP@Poly^RHAPM^, the genome‐wide RNA‐seq was performed on the M1 macrophages treated with PBS and NP@Poly^RHAPM^ (**Figure** **5**A). As illustrated in Figure 5B, the two groups shared 13 632 genes, with 868 and 578 genes only expressed in M1 macrophages treated with PBS and NP@Poly^RHAPM^, respectively. Volcano plots demonstrated that 626 genes were significantly upregulated (red dots) with 371 genes downregulated (green dots) in M1 macrophages treated with PBS and NP@Poly^RHAPM^ (Figure 5C).

To further study the potential signaling pathways affected by NP@Poly^RHAPM^ treatment, a Kyoto Encyclopedia of Genes and Genomes (KEGG) analysis was performed. As demonstrated in Figure 5D, oxidative phosphorylation, reactive oxygen species, NOD‐like receptor signaling pathway, and autophagy were strongly related to the anti‐inflammatory effects of NP@Poly^RHAPM^. Furthermore, Gene ontology (GO) enrichment analysis was performed to investigate the potential effects of NP@Poly^RHAPM^ on biological processes. The results showed that cellular response to DNA damage stimulus, autophagy, and protein phosphorylation, etc., were prominently related to the therapeutic mechanisms of NP@Poly^RHAPM^ (Figure 5E). Likewise, the differential genes were associated with protein‐binding, nucleotide binding, and RNA binding for molecular function, as well as the nucleus, cytoplasm, and mitochondrial on cellular components (Figure S17, Supporting Information).

In addition, the overall upregulation trend of differential genes on autophagy and autophagy of mitochondrion pathway in M1‐type macrophages after NP@Poly^RHAPM^ treatment by Gene Set Enrichment Analysis (GSEA), suggests a potential association between the anti‐inflammatory effect of NP@Poly^RHAPM^ and an augmented autophagic response (Figure 5F; Figure S18, Supporting Information). In addition, cytokine‐mediated signaling pathway, HIF‐1 signaling pathway, and Chemical carcinogenesis ‐reactive oxygen species showed an overall downregulation trend in M1‐type macrophages treated with NP@Poly^RHAPM^ (Figure S19, Supporting Information). Furthermore, the protein‐protein interaction (PPI) network was then built. As shown in Figure 5G, *Il6* and *Tnf* were the core genes in the positive regulation of interleukin‐1 beta production, as well as *Gabarap* was a core gene in the autophagy pathway. The heatmap displays that various autophagy‐related genes such as *Sqstm1*, *Map1lc3a*, *Gabarap*, *Atg14, Atg10*, and *Ulk2* were upregulated after treatment with NP@Poly^RHAPM^ (Figure 5H). Collectively, these data suggest that NP@Poly^RHAPM^ may exhibit anti‐inflammatory effects by directly scavenging excessive reactive oxygen species in M1 macrophages, augmenting subsequent autophagy pathway, and suppressing the secretion of pro‐inflammatory cytokines, which are all beneficial for osteoarthritis treatment.

### 3.6 Protective Effect of NP@Poly^RHAPM^ on Chondrocytes

**Figure 6:**
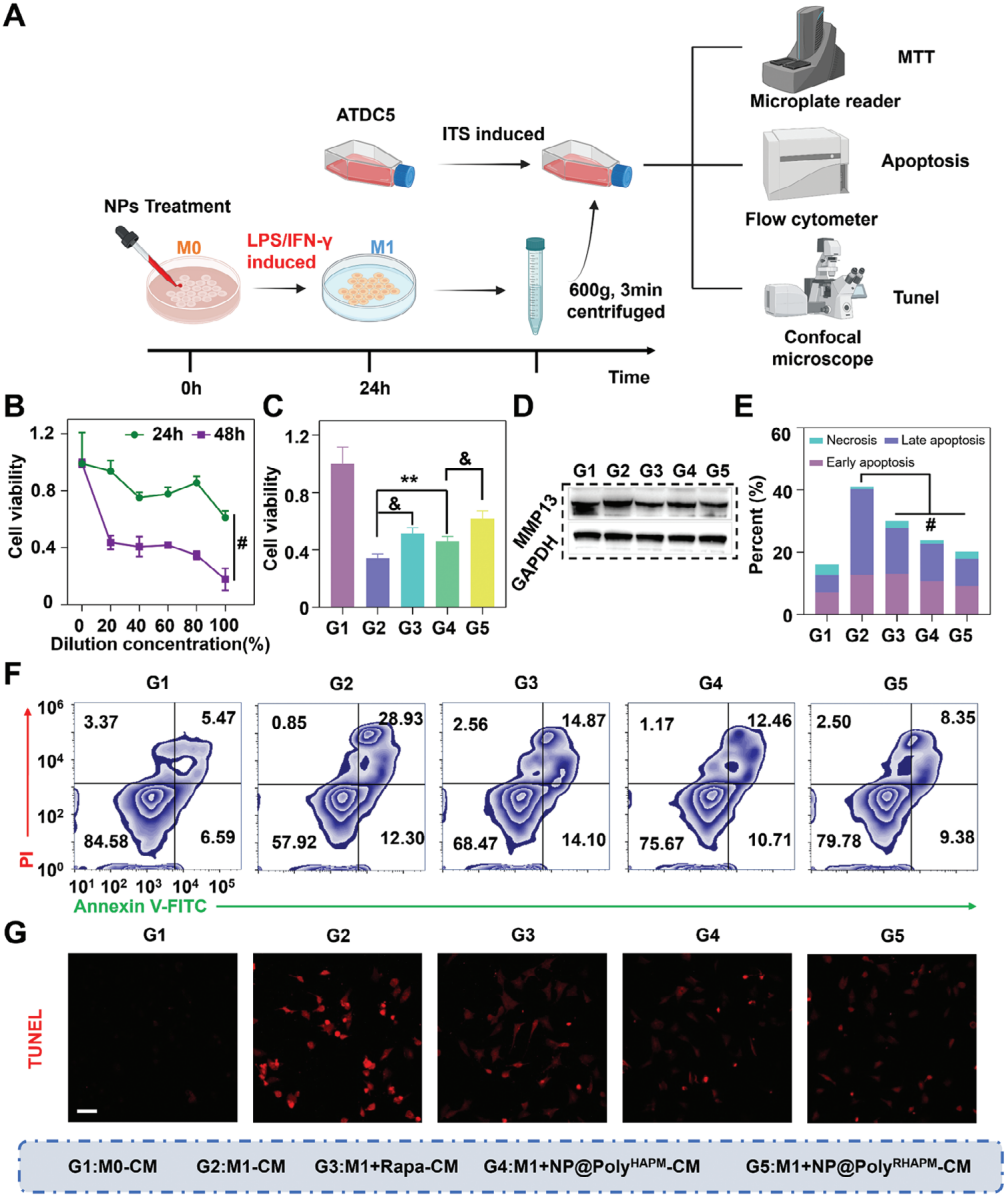
Protective effect of NP@Poly^RHAPM^ on chondrocytes. A) Schematic diagram of macrophage‐conditioned medium co‐cultured with chondrocytes. B) Effect of CM at different time points and different concentrations on the value‐added of ATDC5 chondrocytes. C) Effect of CM of macrophages treated with various drugs on the activity of ATDC5 cells. D) Western blotting showing the expression of MMP13 with various treatment groups. E,F) Apoptosis of ATDC5 cells by CM of activated macrophages treated with various drugs was analyzed using FCM, and the data was quantified. G) CLSM images of ATDC5 cells after different treatments. Scale bar: 50 µm. Data are presented as the mean ± SD (*n = 3*). Statistical significance between every 2 groups was calculated via one‐way ANOVA and two‐way ANOVA tests. ^**^ *p*<0.01, &*p*<0.001 and ^#^ *p*<0.0001.

The effect of NP@Poly^RHAPM^ on ATDC5 chondrocytes was first assessed and the results indicated that the concentrations of Rapa, NP@Poly^HAPM^, and NP@Poly^RHAPM^ used to treat macrophages did not induce inhibition of chondrocyte viability or apoptosis (Figure S20, Supporting Information). Moreover, neither Rapa, NP@Poly^HAPM^ nor NP@Poly^RHAPM^ induced chondrocyte degeneration and significant inflammation after 24 h of incubation with ATDC5 chondrocytes (Figure S21, Supporting Information). These results suggested that NP@Poly^RHAPM^ did not directly affect chondrocytes. The conditioned medium (CM), which was collected after macrophages underwent various treatments, was then co‐incubated for 48 h with ATDC5 chondrocytes that had been stimulated by ITS. The vitality and apoptotic potential of the chondrocytes were then evaluated (**Figure** **6**A). M1 macrophage CM was diluted to different proportional concentration gradients and co‐cultured with ATDC5 cells for 24 and 48 h. The results demonstrated that the activity of ATDC5 cells considerably decreased as the period that CM and chondrocytes were co‐incubated increased. After 48 h of co‐incubation with CM at a 20% dilution concentration, the cell activity was only around 46.6% of what it had been the previous day. The activity of ATDC5 cells likewise showed a substantial decrease trend as CM concentration rose. As the dilution concentration of CM rose from 20% to 80% during a 48 h co‐incubation with chondrocytes, ATDC5 cell activity decreased by 25.9% (Figure 6B), indicating that the time‐ and concentration‐dependent inhibitory impact of M1 macrophage CM on chondrocyte growth.

To further explore the effect of NP@Poly^RHAPM^‐treated macrophages on chondrocyte activity, M1 macrophages were treated with Rapa, NP@Poly^HAPM^, and NP@Poly^RHAPM^ for 24 h, and then cell CM was collected and co‐incubated at a concentration of 20% with ATDC5 for 48 h (Figure 6C). The findings demonstrated that Rapa, NP@Poly^HAPM^, and NP@Poly^RHAPM^ dramatically reversed the inhibitory impact of M1‐CM on ATDC5 cell viability as compared to the M1‐CM group. Noteworthy is the fact that NP@Poly^RHAPM^ therapy decreased the ATDC5 cell death rate from 65.84% in the M1‐CM group to 38.20%. In general, chondrocyte activity can be maintained, and chondrocytes can be shielded from CM inflammatory substances to prevent injury. Additionally, when chondrocytes were incubated with CM for 24 h, NP@Poly^RHAPM^ significantly reduced the relative protein expression of MMP13 in chondrocytes (Figure 6D), indicating that it prevented M1 macrophages from denaturing chondrocytes.

The apoptotic capacity of ATDC5 chondrocytes after treatment with CM from macrophages treated with Rapa, NP@Poly^HAPM^, and NP@Poly^RHAPM^ was then assessed by FCM and TUNEL immunofluorescence. The findings demonstrated that 41.08% of ATDC5 cells underwent apoptosis because of CM collected from M1 macrophages. This value was reduced to 31.24%, 24.01%, or 20.26%, respectively, after treatment with Rapa, NP@Poly^HAPM^, and NP@Poly^RHAPM^ (Figure 6E,F), demonstrating that Rapa, NP@Poly^HAPM^, and NP@Poly^RHAPM^ can greatly limit the apoptotic ability of ATDC5 chondrocytes by CM from M1 macrophages. Additionally, the TUNEL results demonstrated that Rapa, NP@Poly^HAPM^, and NP@Poly^RHAPM^‐treated M1 macrophages successfully reversed the apoptotic state of inflammatory factor‐induced ATDC5 cells (Figure 6G). Further semi‐quantitative analysis of the data revealed that the M1‐CM group's ATDC5 cells underwent 3.7 times less apoptosis when treated with NP@Poly^RHAPM^ (Figure S22, Supporting Information). These findings further confirm that NP@Poly^RHAPM^ has the potential to protect ATDC5 cells from the detrimental effects induced by inflammatory agents.

### 3.7 NP@Poly^RHAPM^ Delays the Progress of OA

**Figure 7:**
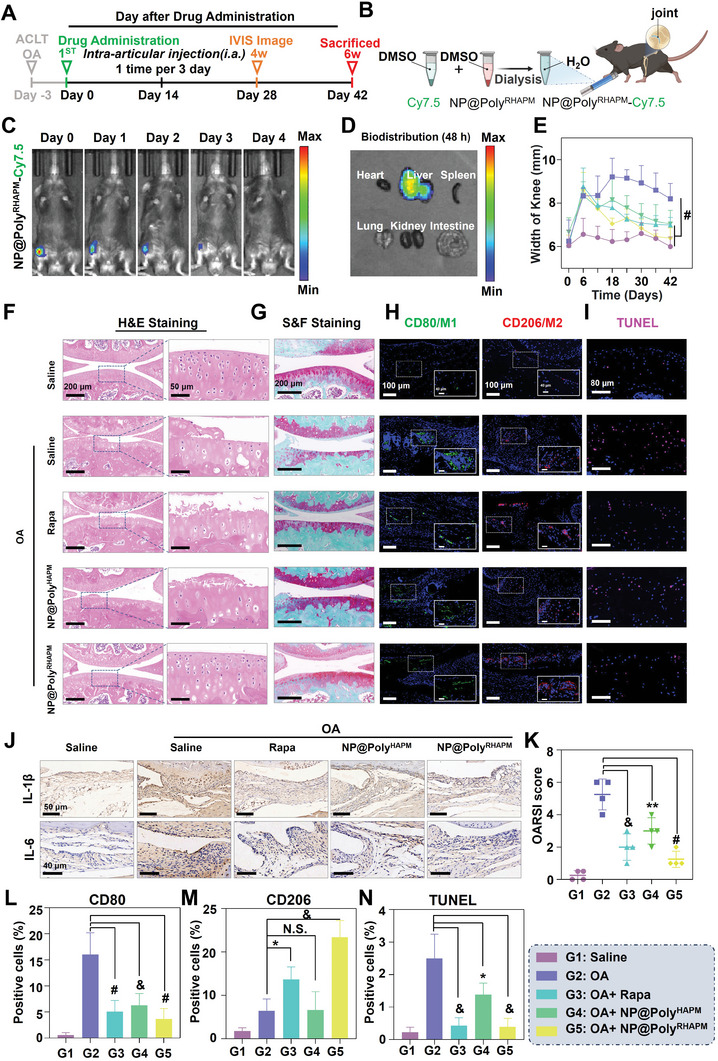
In vivo biodistribution and histological evaluation of NP@Poly^RHAPM^ on ACLT‐induced OA mice. A) Schematic diagram of the in vivo evaluation. B) Illustration of preparing NP@Poly^HAPM^‐Cy7.5, and intra‐articular injection in ACLT‐induced OA mouse. C,D) IVIS images of the biodistribution of NP@Poly^HAPM^‐Cy7.5 and its excretion. E) Knee thickness of mice administrated with various treatment groups. F–I) Representative images of H&E, S&F, and immunostaining of CD80, CD206, and TUNEL after administration of various treatment groups. J) Immunohistochemical analysis of IL‐1β and IL‐6 after administration of various treatment groups. K) OARSI score of the knee joint. L–N) Quantitative analysis of CD80 and CD206 in the synovium, and TUNEL staining in cartilage after administration of various treatment groups. Data are presented as the mean ± SD (*n = 4*). Statistical significance between every 2 groups was calculated via a one‐way ANOVA test. N.S. means no significance, ^*^ *p*<0.05, ^**^ *p*<0.01, &*p*<0.001 and ^#^ *p*<0.0001.

Based on the excellent ROS scavenging and anti‐inflammatory ability of NP@Poly^RHAPM^ in vitro, an in vivo investigation was conducted to assess its therapeutic effect using an OA synovitis model induced by ACLT in mice.^[^ ^30^ ^]^ Specifically, OA mice were treated with different drugs by injection into the joint cavity 3 days after constructing the OA model and then sacrificed after 6 weeks to collect knee tissues for subsequent experiments (**Figure** **7**A). To detect the biodistribution of NP@Poly^RHAPM^, Cy7.5 dyes were encapsulated into NP@Poly^RHAPM^ and traced the bio‐fluorescence in vivo using an IVIS imaging system. As shown in Figure 7B, NP@Poly^RHAPM^ ‐Cy7.5 was obtained by mixing and dissolving Cy7.5 and NP@Poly^RHAPM^ in DMSO, followed by dialysis to filter the organic solvent from the solution, and injecting it into the joint cavity. As shown in Figure 7C, it was demonstrating how the fluorescence intensity of NP@Poly^RHAPM^‐Cy7.5 gradually waned over time, and by day 4, there was no longer any fluorescence to be seen, proving that intra‐articular injections of the medication were necessary every 3 days to keep NP@Poly^RHAPM^ at its half‐life. Additionally, the accumulation of NP@Poly^RHAPM^‐Cy7.5 was found in the liver 48 h after the initial injection, indicating that the liver may be the metabolic pathway of NP@Poly^RHAPM^ (Figure 7D). To assess the effect of NP@Poly^RHAPM^ on the liver, histopathological analysis of the livers of osteoarthritis mice was performed using hematoxylin and eosin (H&E) staining. No significant changes were observed in the H&E‐stained liver tissues following treatment with Rapa, NP@Poly^HAPM^, and NP@Poly^RHAPM^. Similarly, there were no significant alterations in the levels of alanine aminotransferase and aspartate aminotransferase in the serum of mice after treatment with Rapa, NP@Poly^HAPM^, and NP@Poly^RHAPM^, indicating a low hepatotoxicity profile (Figure S23, Supporting Information). Consequently, NP@Poly^RHAPM^ appears to be a highly safe treatment for osteoarthritis therapy. Moreover, during the drug administration period, the claw circumference and knee width of the mice were measured. Notably, in the OA mice group, treatment with Rapa, NP@Poly^HAPM^, and NP@Poly^RHAPM^ resulted in a significant reduction in both knee width and claw circumference (Figure 7E; Figure S24, Supporting Information).

To further assess the therapeutic efficacy of NP@Poly^RHAPM^, the knee joints of mice were stained using H&E and Safranine O‐fast green (S&F). As seen in Figure 7F, the cartilage in the OA group was much more worn out and revealed cartilage layer abnormalities than the saline group. In contrast, Rapa, NP@Poly^HAPM^, and NP@Poly^RHAPM^ successfully slowed down cartilage destruction in OA. Notably, the cells were neatly aligned, and no significant cartilage wear was seen in the NP@Poly^RHAPM^ group. Additionally, the OA group demonstrated significant inflammation, and synovial hyperplasia, vascular opacity formation, and articular bone destruction could all be seen. However, joint inflammation was effectively controlled after treatment with NP@Poly^RHAPM^ (Figure S25, Supporting Information). Moreover, NP@Poly^RHAPM^ significantly decreased the osteoarthritis research society international (OARSI) score and effectively inhibited cartilage matrix degradation in OA (Figures 7G,K). This showed that Rapa, NP@Poly^HAPM^, and NP@Poly^RHAPM^ could effectively reduce joint inflammation and protect cartilage from damage.

The polarization of macrophages in the joint during therapy was also assessed by immunofluorescence staining using M1 and M2 markers. Comparing to the OA group, green fluorescence in OA synovial tissue decreased in the presence of NP@Poly^RHAPM^ treatment, while red fluorescence increased (Figure 7H,L‐M). This indicates that NP@Poly^RHAPM^ effectively inhibited the polarization of M1 macrophages and induced a shift toward M2 macrophage repolarization in the synovial tissue. In addition, previous study has shown that an increase in the mechanical friction of the joints leads to irreversible damage and degradation on the surface of the articular cartilage, which is highly associated with the onset of degenerative diseases such as OA.^[^ ^31^ ^]^ Nanoparticles can serve as a bio lubricant and drug delivery vehicle to ameliorate OA cartilage degradation.^[^ ^32^ ^]^ Zhao and co‐workers demonstrated that nanoparticles with zwitterionic groups possess the capacity to attract a considerable number of free water molecules through ion‐dipole interactions. This interaction results in the formation of a hydrated layer on the surface, facilitating the smooth sliding of hydrated layers over each other and leading to a noteworthy reduction in the coefficient of friction.^[^ ^32^, ^33^ ^]^ Additionally, the synthesis of a bio‐lubricant, pAA‐g‐PEG, involved the coupling of hydrophilic polyethylene glycol (PEG) to polyacrylic acid (PAA). This formulation fixes the thiol terminus, enabling pAA‐g‐PEG to effectively adhere to the cartilage surface.^[^ ^34^ ^]^ The amphoteric material NP@Poly^RHAPM^, characterized by an AST group as the hydrophobic end and thiol‐terminated mPEG_5k_ as the hydrophilic end, holds promise in exhibiting potential lubrication properties in joints. The anti‐inflammatory properties and anticipated improvement in lubrication are beneficial for the treatment of osteoarthritis.

To further understand the impact of NP@Poly^RHAPM^ on the apoptosis of articular chondrocytes, TUNEL fluorescence labeling was employed to measure the fluorescence signal intensity of the cartilage layer. It was found that red fluorescence, indicative of apoptosis, was significantly increased in the OA group but significantly reduced upon NP@Poly^RHAPM^ treatment (Figure 7I,N). Notably, NP@Poly^RHAPM^ also successfully reduced IL‐1β and IL‐6 expression levels in OA synovial tissue. This finding highlights the effective local anti‐inflammatory effects of NP@Poly^RHAPM^ therapy (Figure 7J).

## 4 Conclusion

The modulation of intracellular ROS levels and macrophage polarization shows significant promise for the treatment of OA. In this study, a biodegradable polymer‐based nanoparticle delivery system was developed to scavenge ROS and promote M2 polarization as a therapeutic approach for OA management. The nanoparticle formulation, termed NP@Poly^RHAPM^, was synthesized using a polymer with an oxidation‐sensitive backbone comprising thioketal bonds and an antioxidant component containing multiple conjugated diene unsaturated bonds. Additionally, the nanoparticles were loaded with Rapa. Upon internalization into ROS‐rich M1 macrophages, NP@Poly^RHAPM^ undergoes thioketal bond cleavage, resulting in ROS depletion and the release of AST for further ROS scavenging. Furthermore, NP@Poly^RHAPM^ facilitates the release of encapsulated Rapa, thereby enhancing intracellular autophagy.

In vitro experiments demonstrated that NP@Poly^RHAPM^ upregulated the expression of LC3‐II, an intracellular autophagy marker, promoting the turnover of aged and damaged mitochondria to sustain cellular energy supply and regulate mitochondrial quantity and quality. Concurrently, augmented cellular autophagy inhibited the activation of the NLRP3 inflammasome in macrophages. NP@Poly^RHAPM^ effectively reduced the expression of the inflammatory cytokine IL‐1β by inhibiting NLRP3 inflammasome activation in M1 macrophages. Moreover, NP@Poly^RHAPM^ demonstrated the ability to preserve mitochondrial function in vitro, as evidenced by increased mitochondrial membrane potential, elevated ATP content, and enhanced expression of GSH. Additionally, NP@Poly^RHAPM^ was found to inhibit the polarization of M1 macrophages and promote their conversion to the anti‐inflammatory M2 phenotype. By inhibiting the secretion of inflammatory factors by M1 macrophages, NP@Poly^RHAPM^ effectively protected chondrocytes from the detrimental effects of inflammation. In vivo experiments further revealed that NP@Poly^RHAPM^ facilitated the conversion of synovial M1 macrophages to the M2 phenotype, leading to substantial suppression of synovial inflammation, reduced cartilage destruction, and delayed cartilage degeneration. Overall, this study not only offers a promising strategy for cartilage protection in OA but also holds potential for the treatment of other inflammatory joint diseases. Furthermore, it provides a theoretical foundation for clinical applications aimed at slowing the progression of OA.

## Conflict of Interest

The authors declare no conflict of interest.

## Supporting information

Supporting Information

## Data Availability

The data that support the findings of this study are available from the corresponding author upon reasonable request.
