# Supplementary material for: Reprogramming Macrophage Polarization, Depleting ROS by Astaxanthin and Thioketal‐Containing Polymers Delivering Rapamycin for Osteoarthritis Treatment
Source: Adv Sci (Weinh). 2023 Dec 14;11(9):2305363. doi: 10.1002/advs.202305363 (PMC10916582; doi:10.1002/advs.202305363)
Supplement: Supplementary file 1 — Supporting Information [file ADVS-11-2305363-s001.pdf]

ADVANCED SCIENCE
Open Access

Supporting Information

for *Adv. Sci.*, DOI 10.1002/advs.202305363

Reprogramming Macrophage Polarization, Depleting ROS by Astaxanthin and Thioketal-Containing Polymers Delivering Rapamycin for Osteoarthritis Treatment

Huiyun Li, Yusong Yuan, Lingpu Zhang, Chun Xu, Hailin Xu* and Zhiwei Chen**

Supporting Information

Reprogramming Macrophage Polarization, Depleting ROS by Astaxanthin and Thioketal-Containing Polymers Delivering Rapamycin for Osteoarthritis Treatment

Huiyun Li, Yusong Yuan, Lingpu Zhang, Chun Xu, Hailin Xu*, Zhiwei Chen**

Mr. H. Li
Department of Orthopedic Surgery
The First Affiliated Hospital of University of South China
Hengyang, 421001, Hunan, China

Dr. Y. Yuan
Department of Orthopaedic Surgery, China-Japan Friendship Hospital, No.2 Yinghuayuan East Street, Beijing, 100029, China.
Email: xiaxi@pku.edu.cn

Dr. L. Zhang
Beijing National Laboratory for Molecular Science
State Key Laboratory of Polymer Physics and Chemistry
Institute of Chemistry, Chinese Academy of Science
Beijing 100190, China

Dr. C. Xu
School of Dentistry, The University of Queensland
Brisbane, 4006, Australia
E-mail: chun.xu@uq.edu.au

Prof. H. Xu
Department of Trauma and Orthopedics, Peking University People's Hospital
Diabetic Foot Treatment Center, Peking University People's Hospital, 11th Xizhimen South Street. Beijing, 100044, China
Email: xuhailinfa@163.com

Prof Z. Chen
Department of Orthopedic Surgery
The First Affiliated Hospital of University of South China
Hengyang, 421001, Hunan, China
Email: 430000097010@fsyy.usc.edu.cn

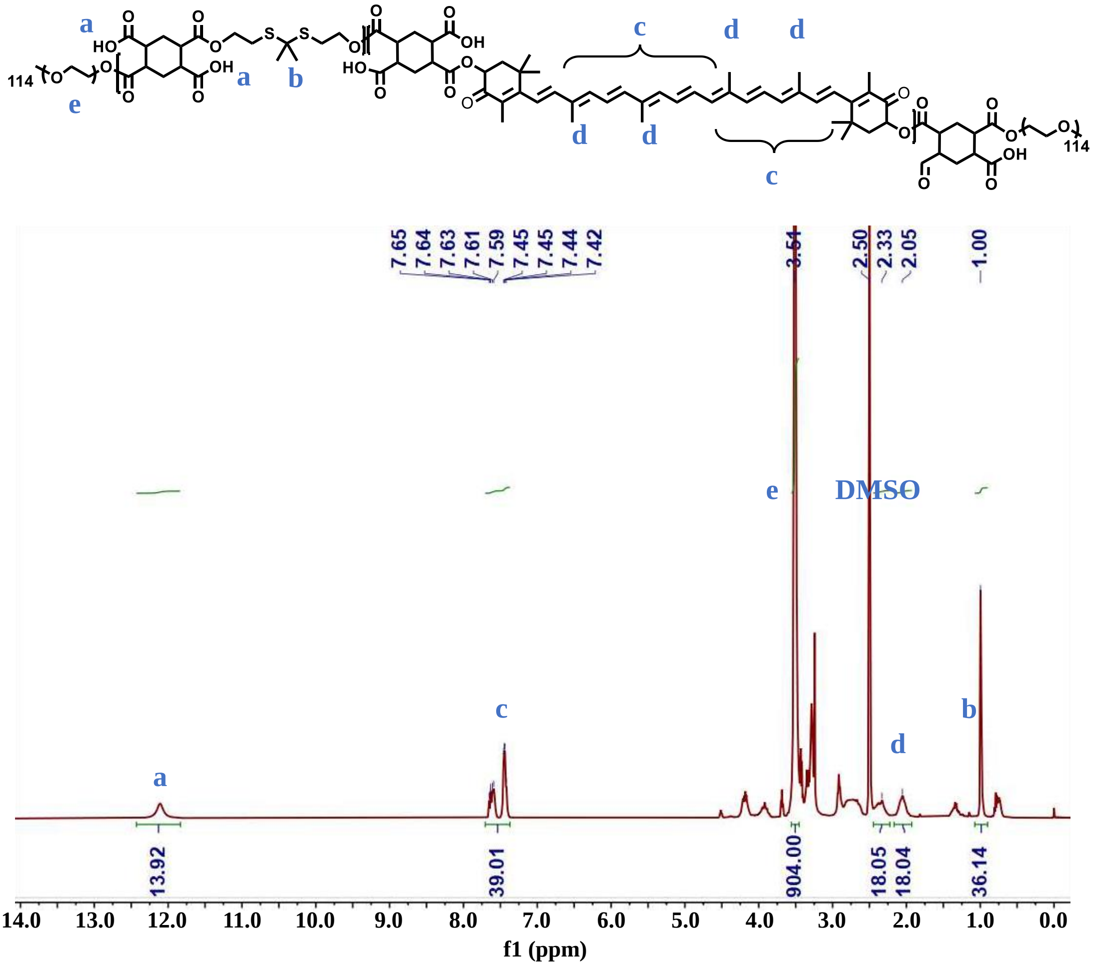

Figure S1. ^{1}H-NMR spectrum of Poly^{HAPM}.

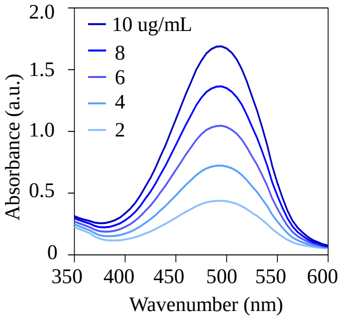

Figure S2. The UV-Vis-NIR absorption spectra of AST at different concentration by using the ultraviolet enzyme labeler.

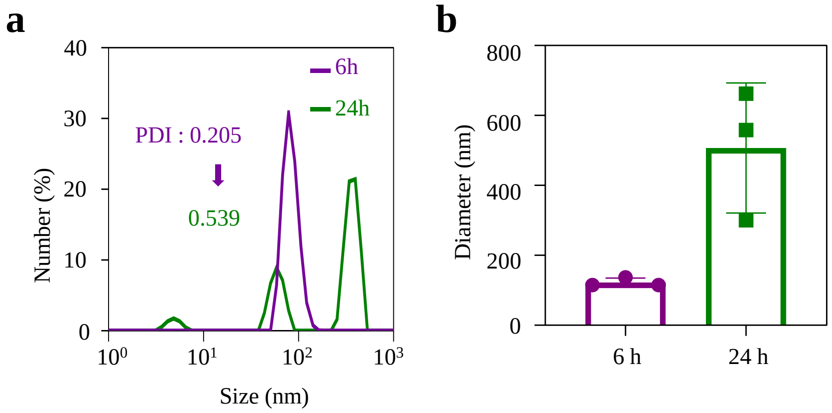

Figure S3. Representative a-b) size distribution and PDI of NP@Poly^{RHAPM} was treated with 10 mM H_2O_2 for 6 and 24 h by DLS.

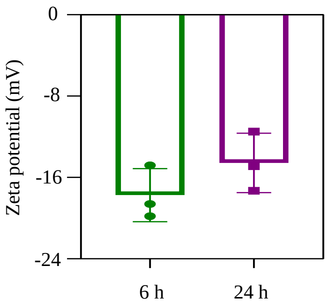

Figure S4. The Zeta potential of NP@Poly^{RHAPM} was treated with 10 mM H_2O_2 for 6 and 24 h by DLS.

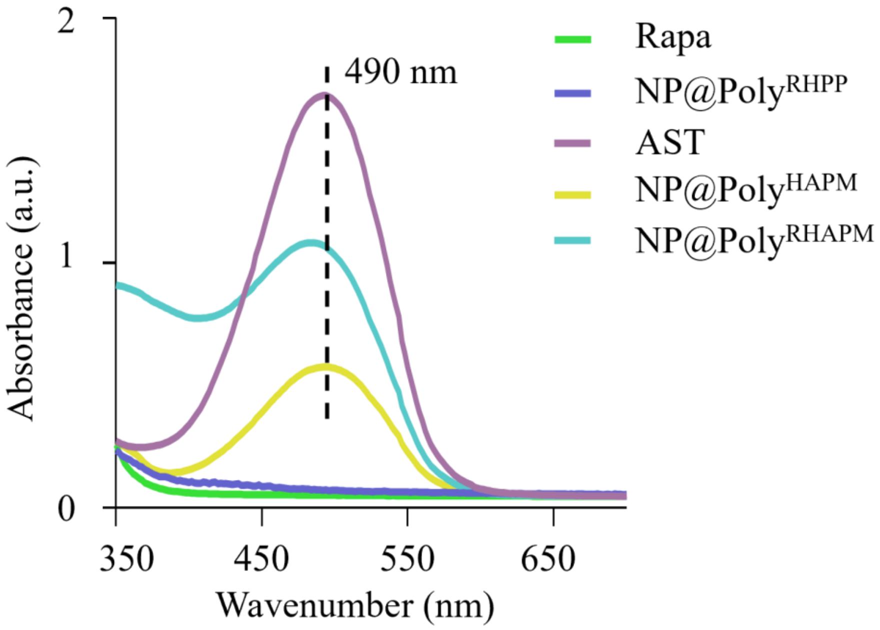

Figure S5. The absorption peak of NP@Poly^{HAPM} and NP@Poly^{RHAPM} by ultraviolet enzyme labeler.

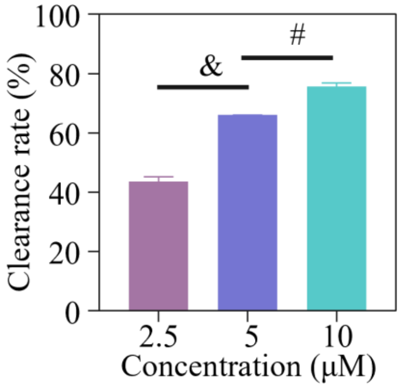

Figure S6. Scavenging H_2O_2 by NP@Poly^{RHAPM} at different polymer concentrations at 4 h. Data are presented as the mean ± SD *(n = 3)*, &*p*<0.001 and #*p*<0.0001.

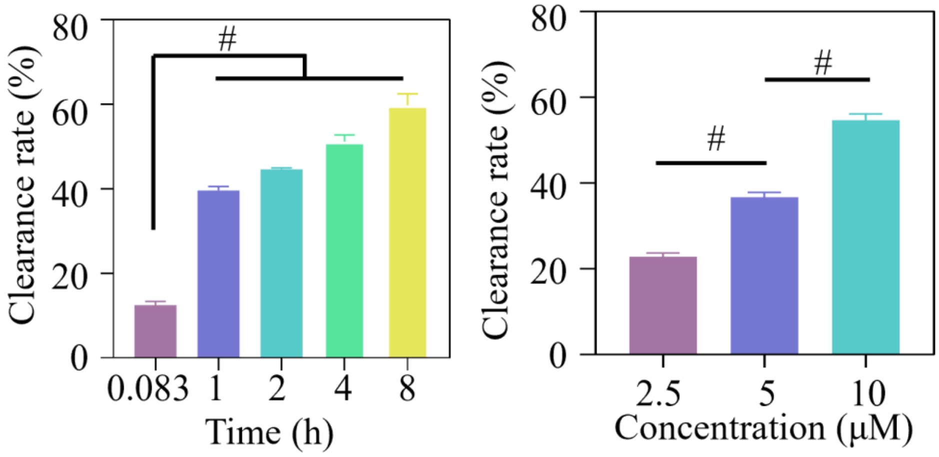

Figure S7. Scavenging H_2O_2 by NP@Poly^{HAPM} at various time points and various concentrations. Data are presented as the mean ± SD *(n = 3)*, #p<0.0001.

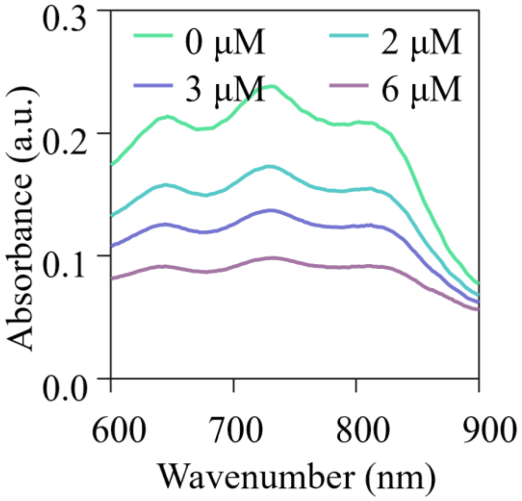

Figure S8. Concentration-dependent scavenging of $ABTS^{+}$ by $NP@Poly^{RHAPM}$ at 4 h.

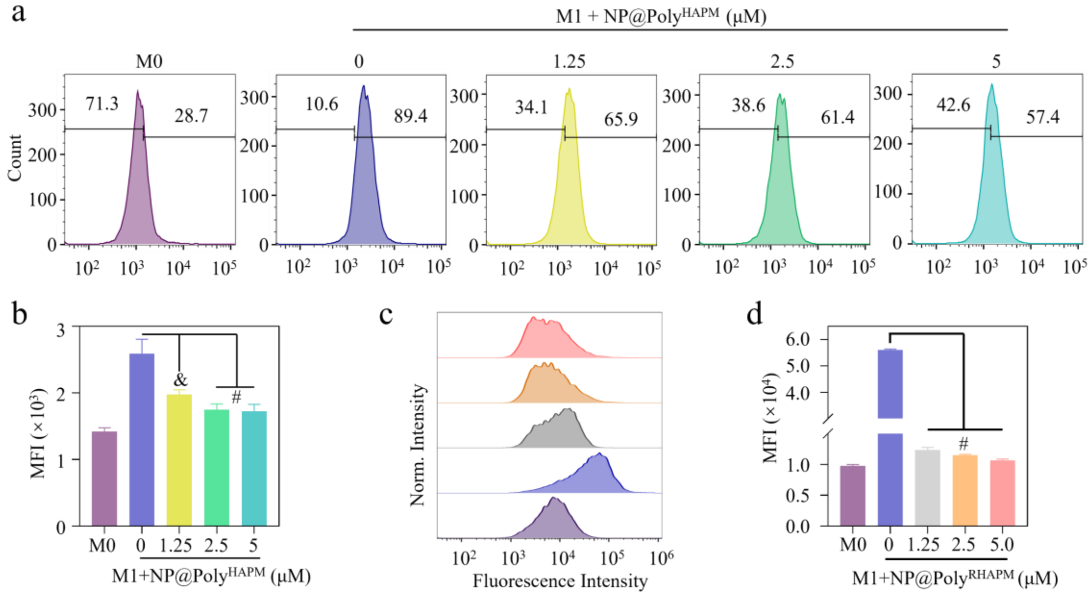

Figure S9. a-b) Quantitative analysis of intracellular ROS levels in macrophages treated with various NP@Poly^{HAPM} concentration using FCM. c-d) Quantitative analysis of intracellular ROS levels in macrophages treated with various NP@Poly^{RHAPM} concentration using FCM. Data are presented as the mean ± SD *(n = 3)*, &*p*<0.001 and #*p*<0.0001.

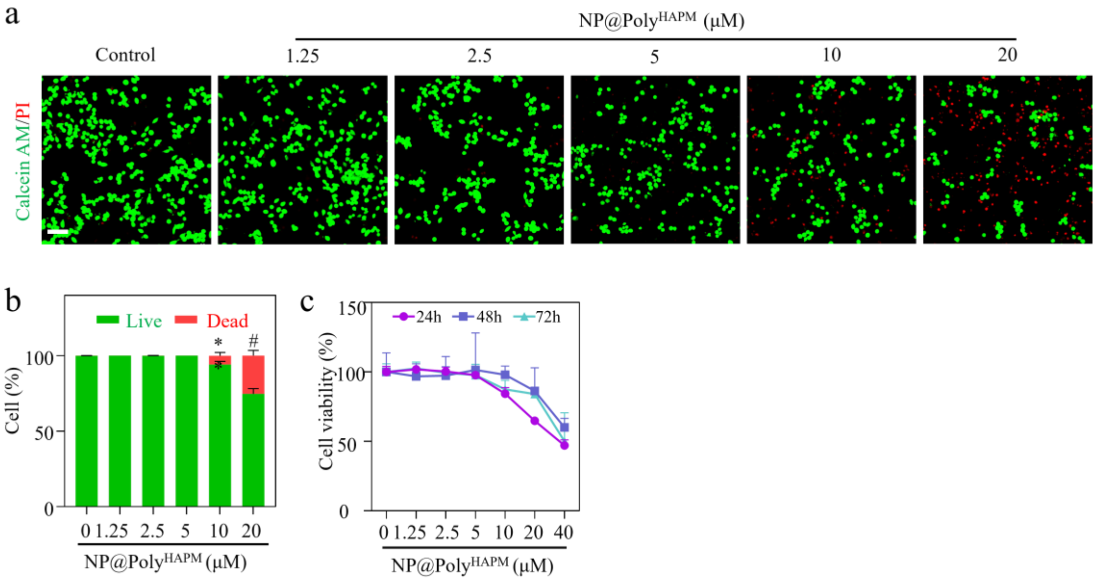

Figure S10. a-b) Live/dead staining of macrophages treated with various concentrations of NP@Poly^{HAPM} for 24 h, and quantitative analysis of the data. Scale bar: 50 μm. c) MTT assay of macrophages treated with various concentrations of NP@Poly^{RHAPM} for 24, 48 and 72 h. Data are presented as the mean ± SD (*n = 3*), $^{**}p<0.01$ and #$p<0.0001$.

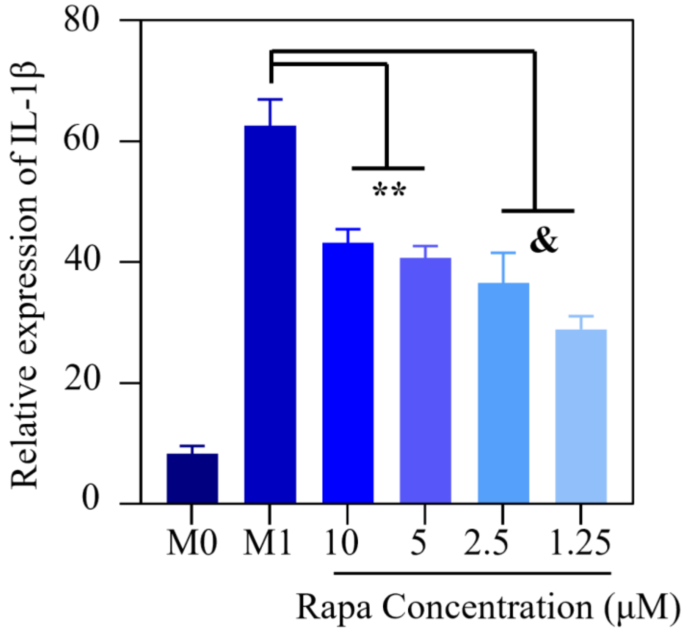

Figure S11. Relative expression of IL-1β on macrophage treated with various Rapa concentration measured by ELISA assay. Data are presented as the mean ± SD (*n* = *3*), ***p*<0.01 and &*p*<0.001.

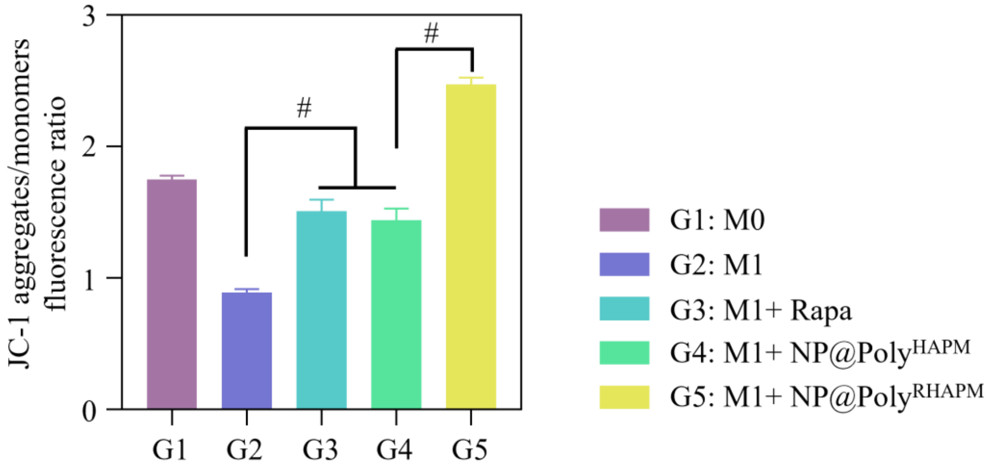

Figure S12. Quantitative analysis of mitochondrial membrane potential within M1 macrophages after different treatment groups. Data are presented as the mean ± SD (*n* = *3*), #*p*<0.0001.

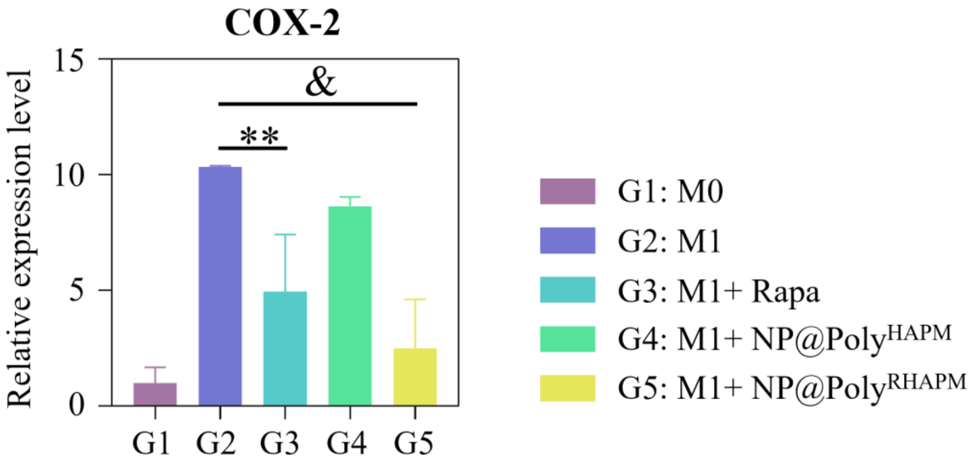

Figure S13. Western blotting showing the expression of COX-2 on macrophage after various treatment groups. Data are presented as the mean ± SD *(n = 3)*, ***p*<0.05 and &*p*<0.001.

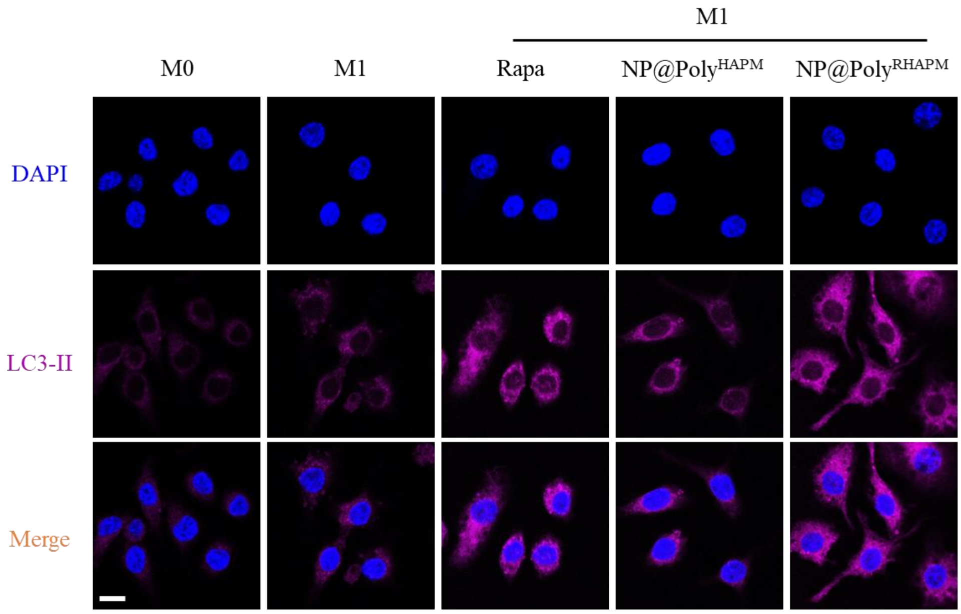

Figure S14. The CLSM images of LC3-II on macrophage after various treatment groups. Scale bar: 10 μM.

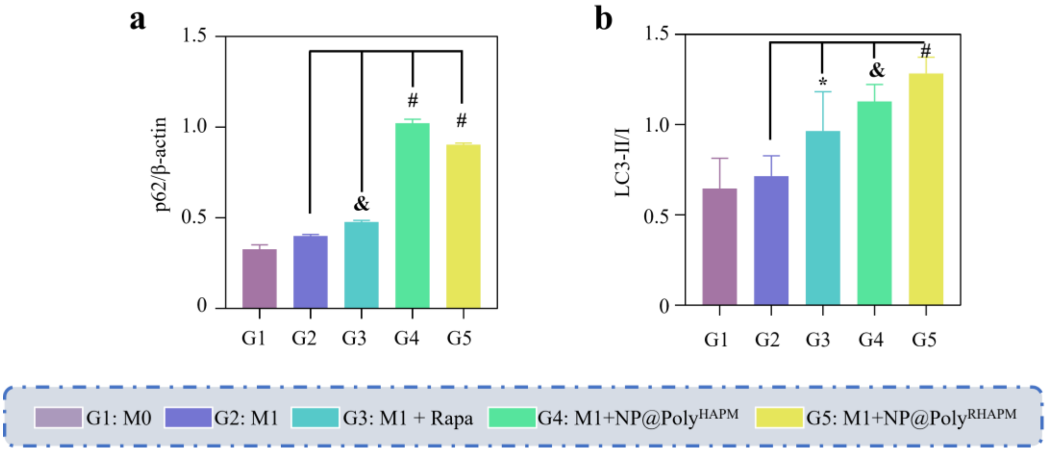

Figure S15. Western blotting showing the expression of a) p62 and b) LC3-II/I on macrophage after various treatment groups. Data are presented as the mean ± SD *(n = 3)*, *p<0.05, &p<0.001 and #p<0.0001.

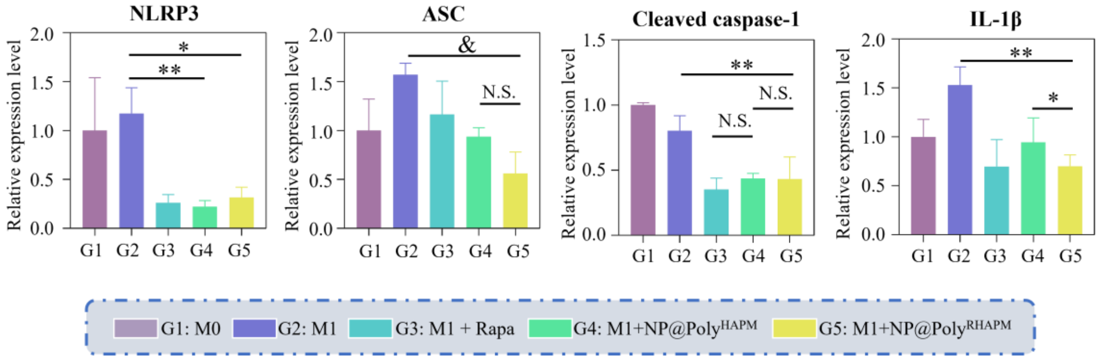

Figure S16. Western blotting showing the expression of NLRP3, ASC, Cleaved caspase-1 and IL-1β on macrophage after various treatment groups. Data are presented as the mean ± SD *(n = 3)*, N.S. means no significance, **p*<0.05, ***p*<0.01 and &*p*<0.001.

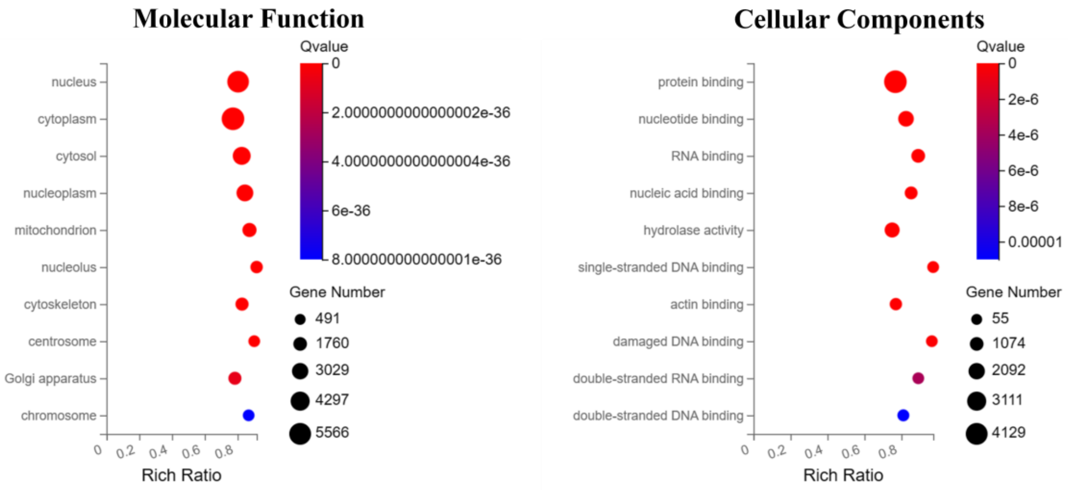

Figure S17. Bubble diagrams of molecular function and cellular components of differentially expressed genes in PBS group and NP@Poly^{RHAPM} treatment group.

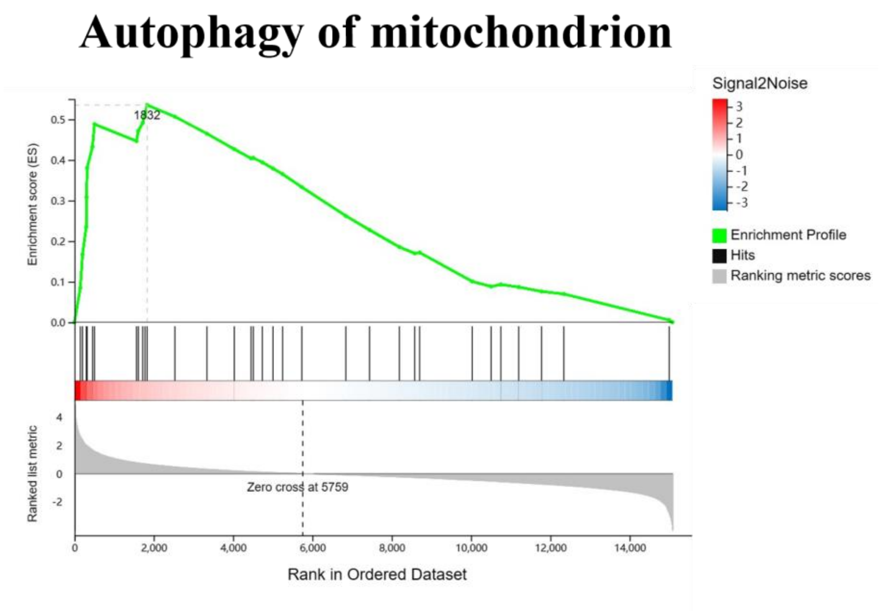

Figure S18. GSEA analysis showing differentially expressed genes in autophagy of mitochondrion in PBS group and NP@Poly^{RHAPM} treatment group.

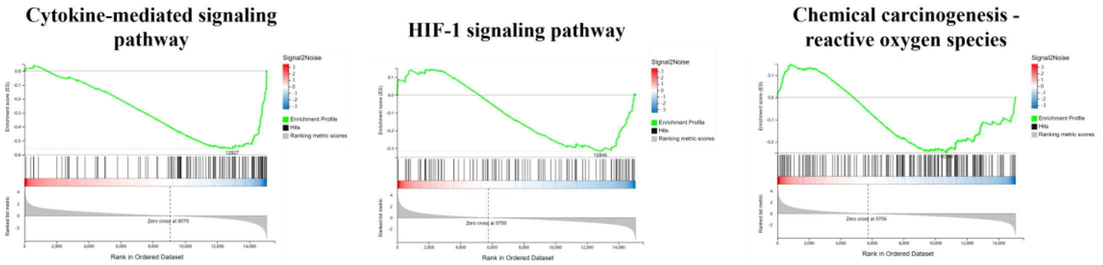

Figure S19. GSEA analysis showing differentially expressed genes in cytokine-mediated signaling pathway, HIF-1 signaling pathway and chemical carcinogenesis - reactive oxygen species in PBS group and NP@Poly^{RHAPM} treatment group.

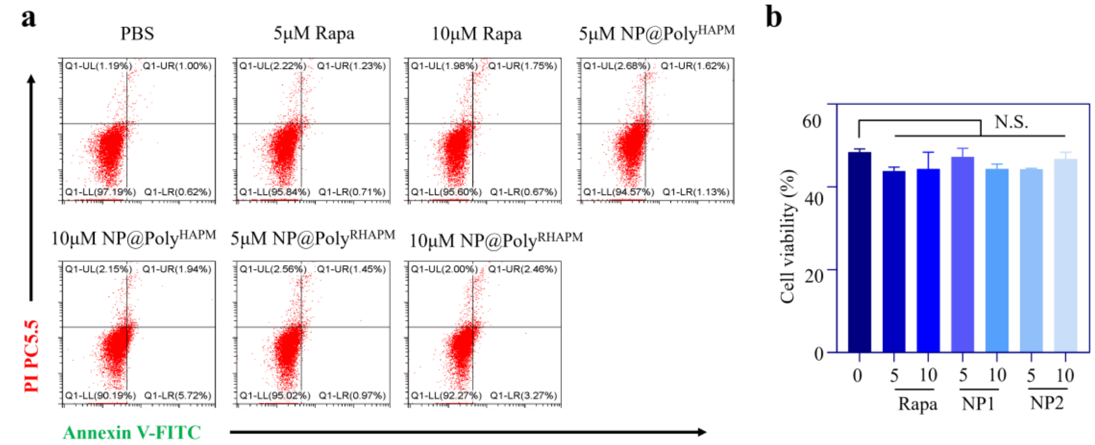

Figure S20. a) Apoptosis of ATDC5 cells after various treatment groups was analyzed using FCM. b) The cell viability of ATDC5 cells after various treatment groups was analyzed using MTT assay. NP1 represents NP@Poly^{HAPM} and NP2 represents NP@Poly^{RHAPM}. Data are presented as the mean ± SD (*n = 3*), N.S. means no significance.

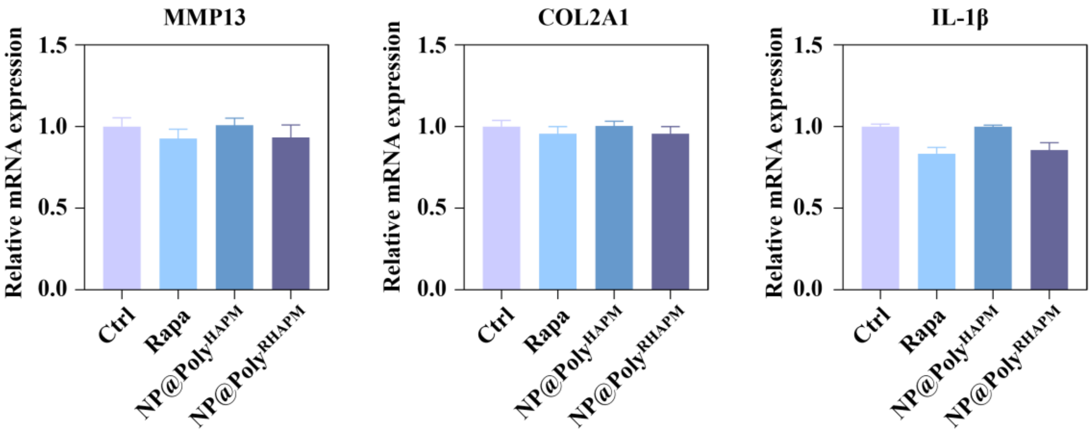

Figure S21. The expression of genes related to chondrocyte inflammation and degradation was assessed by qRT-PCR after treatment with Rapa, NP@Poly^{HAPM} and NP@Poly^{RHAPM}.

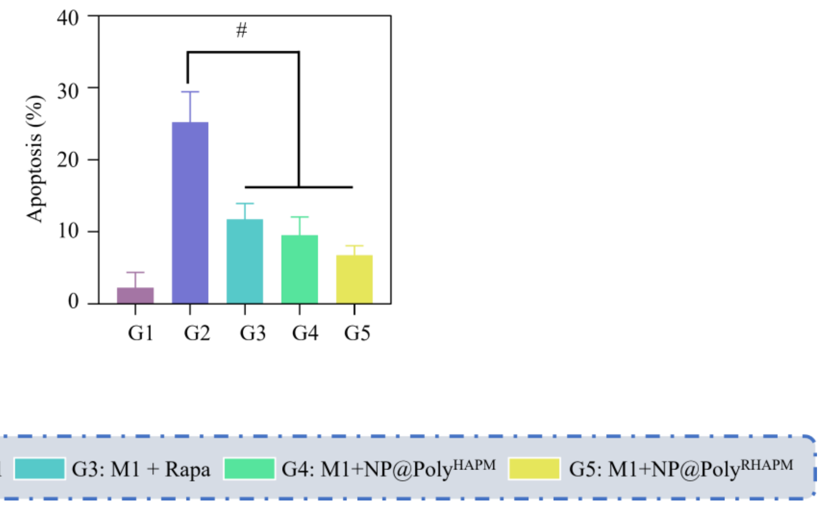

Figure S22. The semi-quantitative analysis of ATDC5 apoptosis rate on TUNEL staining was analyzed. Data are presented as the mean ± SD (n = 3), #p<0.0001.

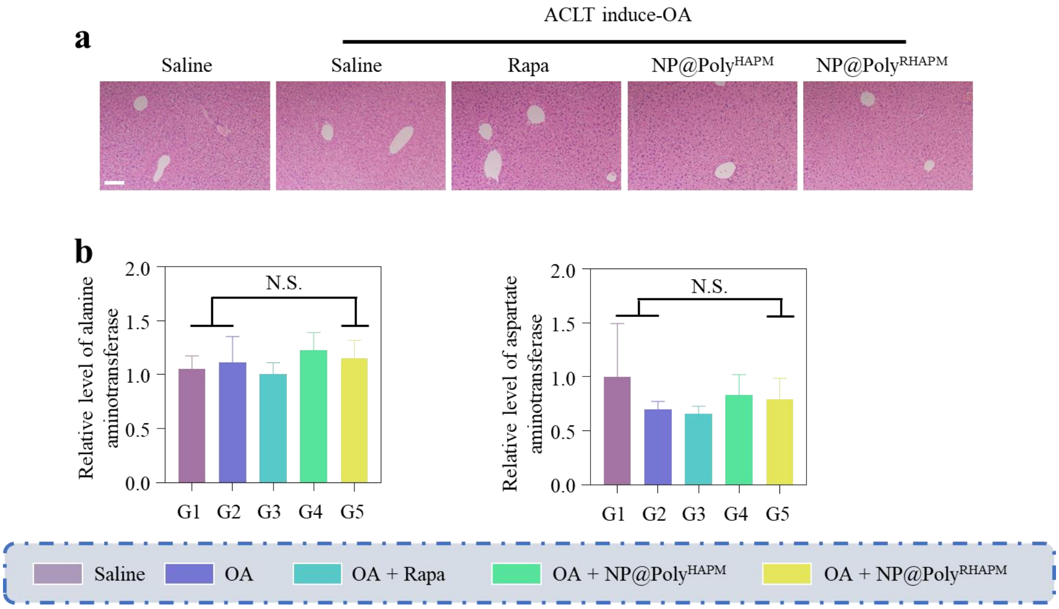

Figure S23. The hepatotoxicity of nanoparticles by analysing the histopathological analysis and liver function index of mice. **a)** The histopathological analysis of liver of OA mice treated with Rapa, NP@Poly^{HAPM} and NP@Poly^{RHAPM} by H&E staining. Scale bars = 100 µm. **b)** The serum liver function index indexes (such as alanine aminotransferase and aspartate aminotransferase) of OA mice treated with Rapa, NP@Poly^{HAPM} and NP@Poly^{RHAPM}. Data are presented as the mean ± SD *(n = 3)*, N.S. means no significance.

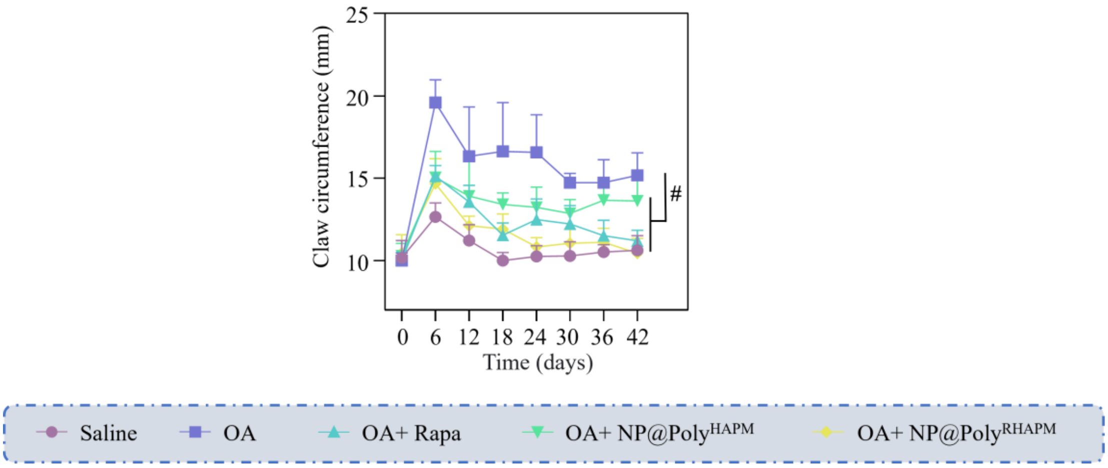

Figure S24. The claw circumference changes of OA mice at various time points after different treatment groups. Data are presented as the mean ± SD (*n* = *3*), #*p*<0.0001.

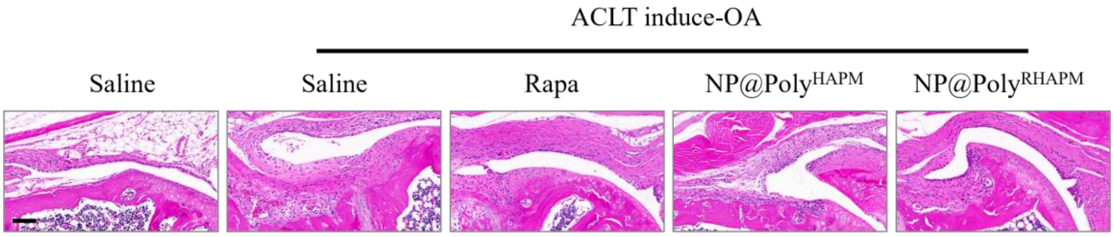

Figure S25. H&E staining of synovial tissue after treatment with various treatment groups, scale bar: 100 μm.
